# In silico evaluation and selection of the best 16S rRNA gene primers for use in next-generation sequencing to detect oral bacteria and archaea

**DOI:** 10.1186/s40168-023-01481-6

**Published:** 2023-03-23

**Authors:** Alba Regueira-Iglesias, Lara Vázquez-González, Carlos Balsa-Castro, Nicolás Vila-Blanco, Triana Blanco-Pintos, Javier Tamames, Maria José Carreira, Inmaculada Tomás

**Affiliations:** 1 Oral Sciences Research Group, Department of Surgery and Medical-Surgical Specialties, School of Medicine and Dentistry, Universidade de Santiago de Compostela, Health Research Institute Foundation of Santiago (FIDIS), C/ Entrerrios s/n, 15872 Santiago de Compostela, Spain; 2 Centro Singular de Investigación en Tecnoloxías Intelixentes and Departamento de Electrónica e Computación, Universidade de Santiago de Compostela, Health Research Institute Foundation of Santiago (FIDIS), Rúa de Jenaro de la Fuente, s/n, 15705 Santiago de Compostela, Spain; 3 Microbiome Analysis Laboratory, Systems Biology Department, Centro Nacional de Biotecnología (CNB)-CSIC, Madrid, Spain

**Keywords:** 16S rRNA gene, Primer, Coverage, Mouth, Bacteria, Archaea, Database

## Abstract

**Background:**

Sequencing has been widely used to study the composition of the oral microbiome present in various health conditions. The extent of the coverage of the 16S rRNA gene primers employed for this purpose has not, however, been evaluated in silico using oral-specific databases. This paper analyses these primers using two databases containing 16S rRNA sequences from bacteria and archaea found in the human mouth and describes some of the best primers for each domain.

**Results:**

A total of 369 distinct individual primers were identified from sequencing studies of the oral microbiome and other ecosystems. These were evaluated against a database reported in the literature of 16S rRNA sequences obtained from oral bacteria, which was modified by our group, and a self-created oral archaea database. Both databases contained the genomic variants detected for each included species. Primers were evaluated at the variant and species levels, and those with a species coverage (SC) ≥75.00% were selected for the pair analyses. All possible combinations of the forward and reverse primers were identified, with the resulting 4638 primer pairs also evaluated using the two databases. The best bacteria-specific pairs targeted the 3-4, 4-7, and 3-7 16S rRNA gene regions, with SC levels of 98.83–97.14%; meanwhile, the optimum archaea-specific primer pairs amplified regions 5-6, 3-6, and 3-6, with SC estimates of 95.88%. Finally, the best pairs for detecting both domains targeted regions 4-5, 3-5, and 5-9, and produced SC values of 95.71–94.54% and 99.48–96.91% for bacteria and archaea, respectively.

**Conclusions:**

Given the three amplicon length categories (100-300, 301-600, and >600 base pairs), the primer pairs with the best coverage values for detecting oral bacteria were as follows: KP_F048-OP_R043 (region 3-4; primer pair position for *Escherichia coli* J01859.1: 342-529), KP_F051-OP_R030 (4-7; 514-1079), and KP_F048-OP_R030 (3-7; 342-1079). For detecting oral archaea, these were as follows: OP_F066-KP_R013 (5-6; 784-undefined), KP_F020-KP_R013 (3-6; 518-undefined), and OP_F114-KP_R013 (3-6; 340-undefined). Lastly, for detecting both domains jointly they were KP_F020-KP_R032 (4-5; 518-801), OP_F114-KP_R031 (3-5; 340-801), and OP_F066-OP_R121 (5-9; 784-1405). The primer pairs with the best coverage identified herein are not among those described most widely in the oral microbiome literature.

Video Abstract

**Supplementary Information:**

The online version contains supplementary material available at 10.1186/s40168-023-01481-6.

## Introduction

The oral microbiota is the second-largest and most diverse in the human body, containing over 700 microbial species [1]. It plays a critical role in the onset and development of two of the most prevalent diseases in humans: dental caries and periodontitis. Both diseases, if left untreated, can lead to tooth loss, edentulism, loss of masticatory function, poor nutrition status, loss of self-esteem, social difficulties, and diminished quality of life [2, 3]. What is more, there is a body of evidence on the association between oral microorganisms and several systemic diseases [4].

The advent of high-throughput next-generation sequencing (NGS) technologies has enabled the characterisation of microbiomes to unprecedented depths that are unachievable with previous methods [5]. These revolutionary techniques enable large-scale projects to be completed in just a few days, or sometimes even hours [6]. The NGS employed most at present—Illumina—can generate sequences with up to 2×300 base pairs (bps) [7]. The NGS of the 16S ribosomal RNA (rRNA) marker-gene amplicons has been widely used to study the oral microbiota [8, 9], allowing the detection of several bacterial and archaeal taxa in both the healthy human mouth and ones with various states of disease [10]. Continuous improvements to the process have recently produced “third-generation sequencing” tools like those from Pacific Biosciences (PacBio) or Nanopore sequencing. These technologies have the objective of generating longer primary read lengths (600–1000 bps) or even the full-length sequence of the 16S rRNA gene (V1–V9 regions) [7].

Further advances in high-throughput sequencing have allowed the development of whole-genome shotgun (WGS) sequencing, which characterises genomes, genes, and genetic features in a sample. Although this technique has several advantages when compared to the 16S rRNA gene sequencing, the latter remains to be widely used in the oral microbiology field mainly due to the rapid processing, the simplicity in analysing the results, and the lower cost [11].

Nevertheless, marker-gene sequencing approaches are also not without shortcomings, with different challenges and pitfalls possible during each step of the gene sequencing workflow [12]. The primer chosen for the polymerase chain reaction (PCR) amplification step can greatly affect the diversity of an investigation’s findings [12, 13]. To amplify a 16S rRNA gene region of interest, “broad-range” (or universal) primers are designed to anneal with the conserved regions flanking the hypervariable zone selected [13]. Although these primers are based on a consensus sequence, some taxa can produce mismatches [12]. Primer bias due to differential annealing can lead to the over- or under-representation of a particular microbial group and, occasionally, even the loss of some groups if there is a poor match with the consensus sequence [14]. As a consequence, using an inadequate primer can lead to questionable biological conclusions [14].

If PCR results in microbial research are to be interpreted satisfactorily, conducting a comprehensive evaluation of a primer’s coverage is essential [15]. The concept of coverage has been defined heterogeneously as follows: the percentage of matches for certain taxonomic ranks [16, 17]; the number of sequences matched by at least one primer [18]; or the proportion of species-level taxonomic entries for each phylum in a database where the prediction is that these will be amplified using a particular primer pair [19]. The literature contains in silico research that analyses the coverage of 16S rRNA gene-targeting primers that are suitable for amplicon sequencing [15–25]. These studies aim to identify the optimum primer pair(s) for sequencing the environmental [16, 17, 20, 21], human [18, 19, 22–24], or combined environmental and human microbiomes [15, 25]. For a few of them, the human mouth was an ecosystem of interest [19, 22, 23], and researchers employed non-oral-specific databases such as Silva [26] or a foregut dataset together with the Ribosomal Database Project (RDP) database [27] for the coverage analysis. The application of phylogenetically diverse databases can, however, produce classification errors since they contain taxonomically misannotated 16S rRNA gene sequences [28]. They also provide different levels of representation for each included environment, leading to substantial variations in the quality of the classifications [29].

Despite the above, to the best of our knowledge, there has been no exhaustive in silico evaluation of the coverage provided by the 16S rRNA gene primers employed in the massive sequencing of mouth specimens using oral-specific databases. Consequently, we aimed to investigate the coverage of primer pairs obtained from examinations of different oral niches in diverse health conditions and ecology studies. To this end, we used two databases containing 16S rRNA gene sequences taken from bacterial and archaeal species found in the human mouth.

## Materials and methods

### Computational search of scientific papers in PubMed and analysis of abstracts using text-mining techniques

We conducted systematic searches of articles in the PubMed database using the R statistical software (version 4.0.3) [30] and the RISmed package (version 2.1.7) [31]. Two searches were conducted for two different purposes: (1) making a list of the 16S rRNA genes primers used to detect and amplify bacteria and archaea in oral samples before massive sequencing; (2) making a list of the archaeal species reported to be inhabitants of the human mouth to create a database of oral-archaea 16S rRNA gene sequences. The groups of words employed in these searches can be found in Additional file 1.

Text-mining techniques were applied to all the downloaded abstracts using the R package tm (version 0.7-7) [32]. Specifically, the abstracts were tokenised, which involved the classification of the words and groups of two or three words contained within them. For purpose 1, publications on the study of bacterial microbiome received a score if their abstracts included terms associated with the oral cavity, and another if they contained terms related to the 16S rRNA gene and its different regions. A further score based on archaea-associated words was used for the articles about the oral archaeome. For purpose 2, the studies identified in the searches seeking to uncover oral archaeal species were rated and assigned an oral and an archaeal word score. The terms used to calculate the scores were the same as those used in the searches. Repeated words were counted only once, meaning that articles with higher scores were purely those with a greater diversity of words. Ultimately, we were left with 129 bacterial and 16 archaeal studies that included the use of at least one different 16S rRNA gene primer, and 53 articles containing information on archaeal species (Table 1). The references of all these papers are included in Additional files 2 and 3.

**Table 1:** Flowchart on the computational search of articles in PubMed and their analysis using text-mining techniques

**Purpose 1. To find 16S rRNA gene primers used to identify bacteria or archaea**			
**Step**	**Description**	**Bacteria**	**Archaea**
1	No. of computational searches in PubMed performed:	2940	5796
2	No. of abstracts and metadata of papers downloaded:	3245	6405
3	No. of papers processed by text-mining techniques:	2939	1687
4	No. of papers with oral score ≥1 and gene score ≥3 (partial reading):	576	44
5	No. of papers reviewed for full-text reading:	323+15^a^	22+12^a^
6	No. of papers with at least one different 16S rRNA gene primer:	129	16
**Purpose 2. To create a list of oral-archaea species**			
**Step**	**Description**		**Archaea**
1	No. of computational searches in PubMed performed:		276
2	No. of abstracts and metadata of papers downloaded:		7548
3	No. of papers processed by text-mining techniques:		6734
4	No. of papers with oral score ≥1 and archaea score ≥3 (partial reading):		200
5	No. of papers reviewed for full-text reading:		60
6	No. of papers with at least one oral archaea species:		53

Papers from “purpose 1” received one score for the oral cavity words included in their abstracts and another for the terms associated with the 16S rRNA gene and its different regions; papers from “purpose 2” received an oral- and an archaeal-word score. In each score, for each different related term included in the abstract, we gave one point with repeated words only counted once (i.e., in a given abstract, the words “oral,” “mouth,” and “periodontitis” appear two, one, and three times so the oral cavity score is equal to three). The terms used to give the punctuations were those used to conduct the searches

^a^Additional publications on the study of the oral microbiota using sequencing were considered for full-text reading; these were previously reviewed for other reasons (*n* = 15) or were found during the search for the oral-archaea species (*n*= 12)

### Primer selection and creating a list of archaeal species found in the oral cavity

In total, we identified 444 16S rRNA gene primers: 203 forward (F), 229 reverse (R), and 12 unidentified (UI). Two hundred and seventy-eight of the primers were procured from the searches on PubMed, being 238 and 37 used for the detection of oral bacteria or archaea, respectively, and three to identify bacteria in the respiratory ecosystem. The remaining 166 were extracted from articles concerning different niches, mainly ecological, described in Klindworth et al. [16]. Of them, 103 corresponded to the bacteria domain, 42 to the archaea domain, and 21 were universal. All 444 primers were assigned a unique identifier based on where they were sourced—“OP” for oral primer and “KP” for Klindworth primer [16]—and their direction (F, R, or UI), followed by a three-digit number (Additional file 4). The 5′–3′ sequences of all 444 primers were then compared to identify repeats, with 75 identified as having the same sequences (Additional file 4). This left us with 369 16S rRNA gene primers with different sequences (with at least one nucleotide difference).

The publications in our final selection were read by two researchers to produce a list of archaeal species found in the human mouth. This gave us 177 different archaea names at the species level (Additional file 5).

### 16S rRNA gene-sequence databases of oral bacteria and archaea for the primer-coverage analysis

#### Modification of an existing 16S rRNA gene-sequence database of oral bacteria

A total of 223,143 amplicon sequence variants (ASVs) of fasta-formatted 16S rRNA gene sequences were included in the Escapa et al. database [33]. The file had been constructed using sequences from the extended Human Oral Microbiome Database (eHOMD) [34] to then conduct a BLASTN search [35, 36] of the National Centre for Biotechnology Information (NCBI) non-redundant nucleotide database [37]. The header line of each sequence had an ASV identifier (from TS000001 to TS223143), followed by a RefSeq [38] or GenBank [39] identifier and an assignment to a seven-level taxonomic hierarchy. The format was as indicated on the DADA2 website [40]. The sequences in the Escapa et al. database [33] were obtained mainly from GenBank [39], and we found that they contain annotation errors that make it impossible to calculate the correct position of the primers within each sequence in the case of a match.

**Fig. 1.**
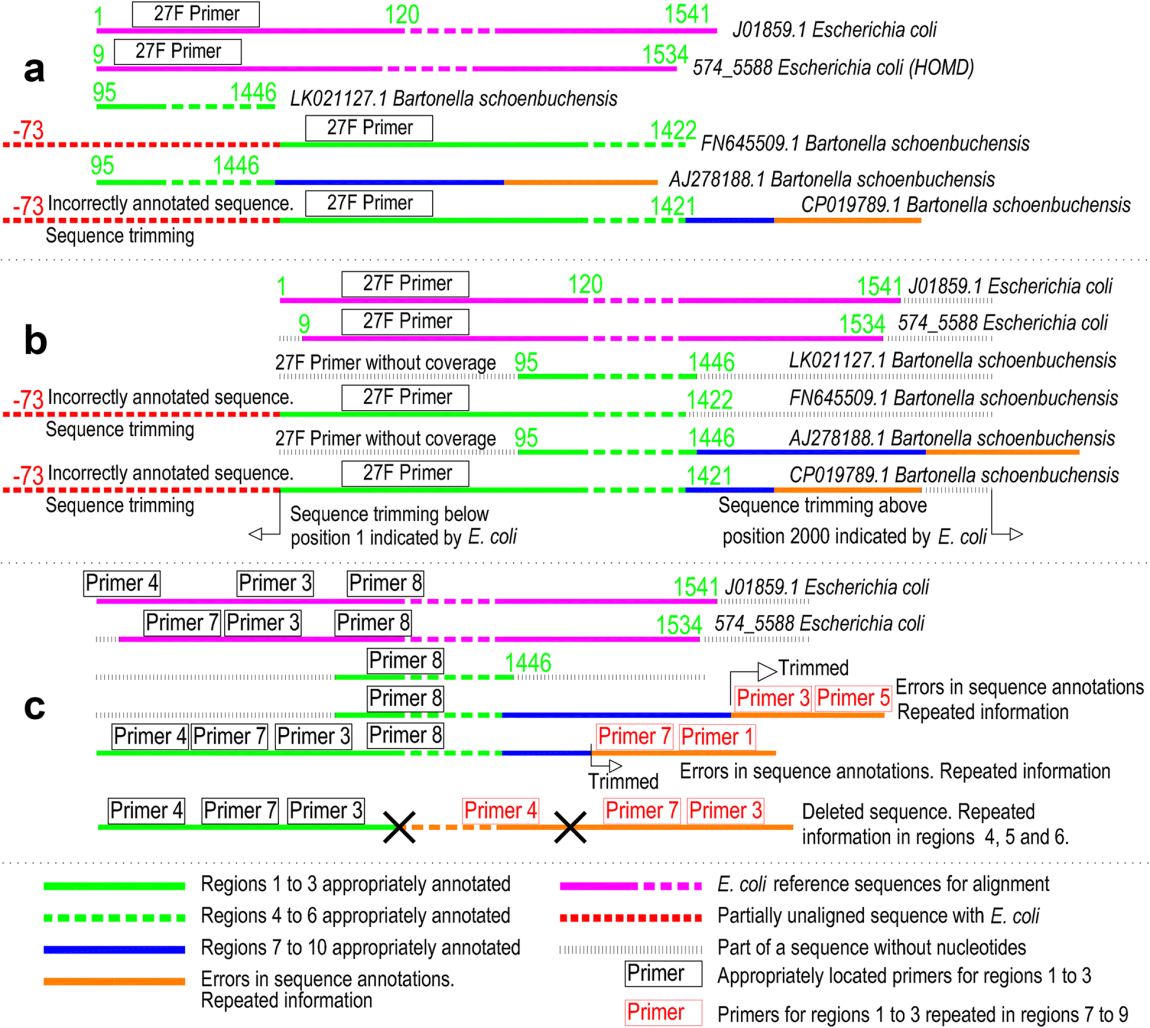
Processing of errors in annotations of oral bacterial and archaeal sequences. **a** Unaligned sequences with missing information at the first and last positions of the 16S rRNA gene, and the presence of redundant information. **b** Alignment of sequences with respect to *E. coli* and trimming of sequences below position 1 and above position 2000 indicated by *E. coli.* **c** Trimming of sequences with redundant information in high regions; and removal of a sequence with repeated information in regions 4, 5, and 6

We developed scripts in Python (version 3.9.0) [41] and Bash (version 5.1) [42] to improve the Escapa et al. database [33]. First, we separated the 16S rRNA gene sequences from ASVs belonging to the same hierarchical level into 769 different fasta files. Second, a species identifier, from SP00001 to SP00769, was attached to all the sequences before the taxonomic hierarchy. Third, sequences from the same hierarchy were aligned simultaneously using clustal Omega [43] against a set of 16S rRNA gene sequences of *Escherichia coli*: three from GenBank and one from HOMD [44]. We installed clustal Omega [43] in the local mode [45] to enable its use with Biopython [46]. The default characteristics were employed to carry out the alignments. Fourth, all the gaps created by clustal Omega [43] were removed, save for those inserted from the start-up to the first nucleotide of each sequence. Fifth, the aligned fasta files were combined in a single file to create a database of fully-aligned *E. coli* ASVs, with position one being the first nucleotide of *E. coli* J01859.1. Lastly, we trimmed the aligned sequences with bases in a lower position than the first nucleotide of J01859.1, as well as those with nucleotides above position 2000 (Fig. 1). The resultant oral-bacteria database is available for consultation in Additional file 6.

#### Creation of a 16S rRNA gene-sequence database of archaea

We searched the NCBI nucleotide database [37] for the complete genomes of the archaeal species found in the human mouth. Along with a script developed in Python [41], these identifiers enabled us to download 193 genomes from RefSeq [38] and eight from GenBank [39].

The script was completed using a free downloadable module, search_16S.py [47], which is based on the algorithm created by Edgar [48]. This allowed us to detect and extract the 16S rRNA gene sequences from the complete downloaded genomes, remove all the repeated sequences, and then store all the variants identified in a fasta file. Prior to use, the search_16S.py algorithm was trained with a RefSeq database containing 16S rRNA gene sequences of archaea stored in the NCBI database [49]. The module and integrating the “The Entrez Programming Utilities (E-utilities)” tool [50] into Biopython [46] meant we could easily and automatically obtain and assign the complete taxonomic rank to the 16S rRNA genes. Biopython [46] also enabled us to access the information of interest requested from the different NCBI databases, such as Taxonomy [51], RefSeq [38], and Genbank [39].

Additionally, the 16S rRNA gene sequences of species without complete genome identifiers in RefSeq [38] or GenBank [39] were searched for in the aforementioned RefSeq archaeal database or, if not found, the Silva database (version 138) [26] or the Genome Taxonomy Database (GTDB) [52]. Finally, all the 16S rRNA gene sequences of the oral-archaeal species were grouped into a single fasta file (Additional file 7).

These sequences were employed to BLASTN [35, 36] against the NCBI non-redundant nucleotide database [37]. Then, we downloaded the 16S rRNA gene sequences with a query coverage ≥98% and a percentage identity ≥99%. The regions aligned with complete genomes were also downloaded using these parameters. Both sequence types were treated as ASVs. We created the oral archaea database using another script developed in Python [41]. This contains 2842 sequences and all the ASVs presenting with a unique identifier with values between AS00001 and AS002842 (Additional file 8). The sequences in the database were aligned with *E. coli* and were improved for the posterior-coverage analysis following the same steps used for the bacteria database (Fig. 1). The definitive oral-archaea database is available for consultation in Additional file 9.

### Coverage ratios of the 16S rRNA gene primers

#### Concept and definition of the coverage ratios calculated for the 16S rRNA gene primers

A sequence was considered covered by a primer when all nucleotides of the primer showed a match with the sequence (mismatches were not allowed). Two types of coverage were defined for the in silico analysis. First, the coverage at the variant level (VC) equated to the percentage of matches of a particular primer concerning the total sequences in the database. In order to minimise the effect on the VC of the absence of information at the ends of sequences, the concept of coverage at the species level (SC) was defined as the percentage of species with matches in at least one of its sequence variants when a particular primer is used.

Matches between the analysed primers and sequences in the databases were evaluated by applying the regular expressions of Python’s regex module [53]. The results were then stored in the Excel format with xlsxwriter [54], which is a Python [41] package that allows the creation and formatting of xlsx files.

#### Selection of primer pairs and analysis of their coverage

All the information related to the coverage analysis of individual primers is included in the Additional file 10.

The individual primers with an SC ≥75.00% were chosen in this stage of the research and all the possible combinations between F and R were identified. We then estimated the mean length between the two positions using the mean position of the first nucleotide of the F primer and that of the last nucleotide of the R primer. The primer pairs had to fulfil two conditions: (1) the mean position of the F primer’s first nucleotide had to be lower than that of the R primer’s last; and (2) the minimum distance between the two means had to be ≥100 nucleotides. The calculated average length was used to classify the primer pairs into one of three categories relating to the mean amplicon lengths: (1) 100 to 300 bps; (2) 301 to 600 bps; and (3) more than 600 bps.

Primer pairs obtained were evaluated against both the bacteria and archaea databases to calculate the coverage parameters defined above. This step enabled us to determine whether a primer pair was bacteria-specific, archaea-specific, or suitable for both domains. A primer pair was assigned the concept of “specific” for bacteria when it had an SC value of 0.00% for archaea, and it was “specific” for archaea when it had an SC value of 0.00% for bacteria. A primer pair was considered “non-specific” if it showed SC values >0.00% in both domains.

Taxa covered and not covered by the different primer pairs evaluated were described, the latter being those with 16S rRNA sequences showing at least one mismatch with the tested primer pair.

## Results

Of the 369 individual primers, 178 (103 F, 75 R) and 50 (33 F, 17 R) showed some coverage value for only bacteria or only archaea, respectively. One hundred and twenty-four (30 F, 94 R) were able to detect both oral-bacterial and archaeal species, while 17 (9 F, 8 R) were not able to detect any such organisms.

The metrics obtained using the two databases for individual primers as well as all the possible combinations of primer pairs are included in Additional files 11-13. The bacterial and archaeal SC values were ≥ 75.00% in 148 (67 F, 81 R) and 65 (19 F, 46 R) individual primers, respectively. After applying the primer-pair formation criteria, 3993 bacterial and 645 archaeal combinations were possible. Of these, 156 were repeated primer pairs in both domains, and the rest (i.e., 3837 and 489) were obtained exclusively when searching for bacterial or archaeal primer pairs.

### Evaluation of 16S rRNA gene primer pairs for the detection of oral bacteria, archaea, and both domains

Results obtained in the analysis of the individual primers are included in the Additional file 10.

#### Bacteria-specific primer pairs

The pair’s analysis revealed that 3218 of the 3837 bacteria-specific primer pair candidates had an archaeal VC and SC of 0.00%. On the other hand, 619 had some coverage value for oral archaea (archaea VC range = 52.25–0.04%; archaea SC range= 70.62–0.52%). Relative to the mean lengths of the generated amplicons: 840 primer combinations had bps of 100 to 300; 1374 from 301 to 600; and 1623 more than 600.

In the first amplicon length category, 139 pairs had bacterial SC values ≥ 95.00% (bacterial SC range = 99.09–95.19%), while 33 also had an archaeal SC of 0.00%. The latter were used to amplify gene regions 3-4 or 5-7 and had bacterial SC values ranging from 97.92% to 95.58%, which meant that 16 to 34 oral bacterial species were not covered. For most of these, the mean read length of their amplicons was around 186 (range = 189–182). However, the pair OP_F009-OP_R030 from region 5-7 stood out, with a mean read length of 297 and a bacterial SC value of 96.88%, which left only 24 bacterial species from the oral cavity being not covered by this pairing.

Sixty-eight primer pairs in the second amplicon length category had bacterial SC values ≥ 95.00% (range = 98.83–95.06%). Of these, 45 did not amplify any archaeal species and could therefore be treated as bacteria-specific. Their bacterial SC values also ranged from 98.83% to 95.06%, meaning that between nine and 38 species were not covered. In addition, these pairs targeted gene regions 3-5, 3-6, or 4-7, and had maximum (max.) and minimum (min.) mean read lengths of 566 and 454, respectively. Of those with the longest mean amplicon lengths, the pairs providing the best coverage were, in order: KP_F051-OP_R030; OP_F021-OP_R030; KP_F048-OP_R073; KP_F051-KP_R053; OP_F021-KP_R053; and OP_F050-OP_R073 (bacterial SC range = 98.83–96.23%; mean read length range = 566–546). These pairs, which amplified regions 3-6 or 4-7, did not cover between nine and 29 oral-bacteria species.

Lastly, 20 primer pairs with mean amplicon lengths >600 bps had bacterial SC values ≥ 95.00% (range = 97.14–95.06%), while 17 also had an archaeal SC value of 0.00%. These pairs had the same bacterial SC range and left between 22 and 38 species uncovered. All of them targeted gene region 3-7 and had max. and min. mean read lengths of 772 and 732, respectively. The primers with the best balance between the mean read length and the coverage were as follows: KP_F048-KP_R074 (bacterial SC = 97.01%; mean read length = 767); and OP_F050-KP_R074 (bacterial SC = 96.36%; mean read length= 766). There were, however, interesting options for the bacteria-specific pairs with mean amplicon lengths >1000 bps and bacterial SC values ≥ 90.00% (bacterial SC range = 93.37–90.64%; mean read length range= 1066–1059). In this sense, the pairs KP_F048-KP_R060, KP_F048-KP_R076, and KP_F048-OP_R121 from region 3-9 had mean read lengths of 1061, 1060, and 1060, respectively, and bacterial SC values of 93.37%; these pairings left a total of 51 oral bacteria species uncovered.

For each mean amplicon-length category, we selected at least one primer pair suitable for detecting only bacteria (archaeal SC= 0.00%) and which targeted distinct 16S rRNA gene regions (Table 2). The pairs had to have a bacterial SC ≥ 90.00% and were chosen based on their coverage and mean amplicon lengths. The VC results of these selected primers are detailed in the Additional file 14.

**Table 2:** Selected primer pairs for detecting oral bacteria in different amplicon-length categories

		Bacteria					Archaea				
ALC (bps)	Primer pair	Gene region	SC (%)	Covered	Not covered	Mean length	Gene region	SC (%)	Covered	Not covered	Mean length
100–300	KP_F048-OP_R043	3-4	97.92	753	16	183	-	0.00	0	194	0
	OP_F098-OP_R119	4-5	94.54	727	42	289	-	0.00	0	194	0
	OP_F066-KP_R040	5-6	90.25	694	75	142	-	0.00	0	194	0
	OP_F009-OP_R030	5-7	96.88	745	24	297	-	0.00	0	194	0
	OP_F101-OP_R030	6-7	93.63	720	49	164	-	0.00	0	194	0
	KP_F061-KP_R074	6-7	91.94	707	62	206	-	0.00	0	194	0
301–600	KP_F048-KP_R031	3-5	97.53	750	19	455	-	0.00	0	194	0
	KP_F048-OP_R073	3-6	96.88	745	24	547	-	0.00	0	194	0
	KP_F048-OP_R050	3-6	90.25	694	75	579	-	0.00	0	194	0
	KP_F051-KP_R041	4-6	90.77	698	71	411	-	0.00	0	194	0
	KP_F051-OP_R030	4-7	98.83	760	9	566	-	0.00	0	194	0
	OP_F116-KP_R060	7-9	94.02	723	46	308	-	0.00	0	194	0
>600	KP_F048-OP_R030	3-7	97.14	747	22	733	-	0.00	0	194	0
	KP_F048-KP_R074	3-7	97.01	746	23	767	-	0.00	0	194	0
	KP_F048-KP_R060	3-9	93.37	718	51	1061	-	0.00	0	194	0
	KP_F056-KP_R077	4-9	91.94	707	62	845	-	0.00	0	194	0

Species coverage was estimated as the number of species with at least one match in an ASV divided by the number of species included in the database. Our bacterial and archaeal databases contained 769 and 194 species, respectively, each of which had between one and 4000 ASVs. The location of the first and last nucleotides of each primer within each sequence with a match was calculated and the mode values for these positions were determined. If there was more than one mode for a position, we chose the one closest to the mean position value. As all the sequences in the two databases were aligned with the 16S rRNA *E. coli* gene, the mode values obtained for each primer enabled us to allocate them to one of the gene regions defined for that organism by Baker et al. [55]. The reference sequence utilised had 1542 bps distributed in 10 conserved (C1–C10) and nine hypervariable regions (V1–V9). The sequences of these selected primer pairs are described in Additional file 15

*ALC* amplicon length category, *bps* base pairs, *F* forward, *KP* Klindworth primer, *OP* oral primer, *R* reverse, *SC* coverage at the species level

Additional file 16 includes the oral species not detected by the primer pairs that were found to achieve a bacterial SC ≥ 95.00% and an archaeal SC= 0.00%, as well as those named previously or included in Table 2 that produced a bacterial SC ≥ 90.00% and an archaeal SC= 0.00%.

#### Archaea-specific primer pairs

Of the 489 primer pairs that were specifically archaea-domain candidates, 359 simultaneously had a bacterial VC and an SC of 0.00%. Conversely, 130 had some coverage value for oral bacteria (bacterial VC range= 9.98–0.01%; bacterial SC range= 74.64–0.13%). Classification of all the pairs based on their mean amplicon lengths revealed that: 77 had 100 to 300 bps; 209 had 301 to 600; and 203 had more than 600.

Twelve primer pairs in the 100–300 bps category had archaeal SC values ≥ 95.00% (range= 98.45–95.36%). Of these, eight had bacterial SC values of 0.00% and should therefore be defined as archaea-specific: OP_F066-KP_R013; KP_F059-KP_R013; KP_F016-KP_R002; KP_F018-KP_R003; OP_F066-KP_R006; KP_F018-OP_R102; KP_F059-KP_R006; and KP_F018-KP_R002. Their archaeal SC ranged from 95.88% to 95.36%, their max. and min. read lengths from 275 to 144, and they were employed to amplify gene regions 3 or 5-6. The use of these pairs would leave between eight and nine oral-archaeal species uncovered.

Nineteen primer pairs in the second amplicon length category had archaeal SC values ≥ 95.00% (archaeal SC range = 97.42–95.36%). Among these, nine also had a bacterial SC value of 0.00%: KP_F018-KP_R031; KP_F018-KP_R032; KP_F018-KP_R035; KP_F018-OP_R020; KP_F018-OP_R070; KP_F020-KP_R006; KP_F020-KP_R013; KP_F016-KP_R032; and OP_F114-KP_R006. These targeted gene regions were 3-5 or 3-6 and had mean amplicon lengths of 551 to 414 bps. The pairs covered 95.88% to 95.36% of the oral archaea species in our database, leaving between eight and nine uncovered.

Only one primer pair in the >600 bp category had an SC value ≥ 95.00% in the archaea database: OP_F114-KP_R013. Interestingly, it also had a bacterial SC value of 0.00%. This pair was used to amplify gene region 3-6, had a mean length of 679 bps, and left eight archaeal species uncovered. We obtained 27 pairs of primer combinations with an archaeal SC ≥ 90.00%, a bacterial SC of 0.00%, and a mean length >679 bps, 10 of which were longer than 1100 bps (max. mean length= 1131; min. mean length= 681). Of these, the best balance between coverage and the mean amplicon length was found in: KP_F016-KP_R066; KP_F016-KP_R063; KP_F018-KP_R066; and KP_F018-KP_R063. Their archaeal SC was 92.78% for the first two pairs and 93.81% for the second two, leaving 14 or 12 species, respectively, uncovered. All of these pairs targeted gene region 3-9 and had, in order, mean amplicon lengths of 1129, 1128, 1119, and 1118.

At least one primer pair suitable for detecting only archaea (bacterial SC= 0.00%) in the different 16S rRNA gene regions was selected (Table 3). They had to present an archaeal SC ≥ 90.00% and were chosen based on both their coverage and mean amplicon lengths. The VC results of these selected primers are detailed in the Additional file 14.

**Table 3:** Selected primer pairs for detecting oral archaea in different amplicon-length categories

		Bacteria					Archaea				
ALC (bps)	Primer pair	Gene region	SC (%)	Covered	Not covered	Mean length	Gene region	SC (%)	Covered	Not covered	Mean length
100–300	KP_F018-KP_R002	-	0.00	0	769	0	3	95.88	186	8	144
	KP_F016-KP_R003	-	0.00	0	769	0	3	94.85	184	10	158
	OP_F066-KP_R013	-	0.00	0	769	0	5-6	95.88	186	8	275
301–600	KP_F018-KP_R032	-	0.00	0	769	0	3-5	95.88	186	8	414
	KP_F018-OP_R073	-	0.00	0	769	0	3-5	90.72	176	18	510
	KP_F020-KP_R013	-	0.00	0	769	0	3-6	95.88	186	8	542
	OP_F114-KP_R007	-	0.00	0	769	0	3-6	92.78	180	14	557
	KP_F022-OP_R016	-	0.00	0	769	0	5-9	92.78	180	14	490
	KP_F022-KP_R063	-	0.00	0	769	0	5-9	91.75	178	16	585
>600	OP_F114-KP_R013	-	0.00	0	769	0	3-6	95.88	186	8	679
	KP_F018-KP_R063	-	0.00	0	769	0	3-9	93.81	182	12	1118
	KP_F016-KP_R063	-	0.00	0	769	0	3-9	92.78	180	14	1128
	OP_F066-OP_R016	-	0.00	0	769	0	5-9	92.78	180	14	624

Species coverage was estimated as the number of species with at least one match in an ASV divided by the number of species included in the database. Our bacterial and archaeal databases contained 769 and 194 species, respectively, each of which had between one and 4000 ASVs. The location of the first and last nucleotides of each primer within each sequence with a match was calculated and the mode values for these positions were determined. If there was more than one mode for a position, we chose the one closest to the mean position value. As all the sequences in the two databases were aligned with the 16S rRNA *E. coli* gene, the mode values obtained for each primer enabled us to allocate them to one of the gene regions defined for that organism by Baker et al. [55]. The reference sequence utilised had 1542 bps distributed in 10 conserved (C1–C10) and nine hypervariable regions (V1–V9). The sequences of these selected primer pairs are described in Additional file 15

*ALC* amplicon length category, *bps* base pairs, *F* forward, *KP* Klindworth primer, *OP* oral primer, *R* reverse, *SC* coverage at the species level

Additional file 17 contains the species not covered by the pairs that achieved an archaeal SC ≥ 95.00% and a bacterial SC= 0.00%, as well as those named above or included in Table 3 with an archaeal SC ≥ 90.00% and a bacterial SC= 0.00%.

#### Bacterial and archaeal primer pairs

The 156 primer combinations that were candidates for detecting both bacteria and archaea had bacterial and archaeal SC values ranging from 98.57% to 75.42% and 99.48% to 73.71%, respectively. Our classification of the combinations based on their mean amplicon lengths revealed that: 40 pairs had between 100 and 300 bps; 42 from 301 to 600; and 74 more than 600.

Ten pairs in the 100–300 bps category had bacterial and archaeal SC values ≥ 95.00%, with a range from 95.97% to 95.32% for the former and from 99.48% to 97.94% for the latter. The max. mean length was 288 bps and the min. 284. All the pairs targeted gene region 4-5 and had been assigned the following identifiers: KP_F020-KP_R031; KP_F020-OP_R070; KP_F020-KP_R032; KP_F020-KP_R035; KP_F020-OP_R020; KP_F020-KP_R038; KP_F020-OP_R010; KP_F020-OP_R014; KP_F020-OP_R036; and KP_F020-OP_R048. The number of bacterial species that were not covered by these pairs ranged from 31 to 36; for the oral-archaeal species, this range was one to four.

Two primer pairs in the 301–600 bps category had bacterial and archaeal SC estimates ≥ 95.00%: OP_F114-OP_R070 (bacterial SC= 95.58%; archaeal SC= 98.45%); and OP_F114-KP_R031 (bacterial SC= 95.71%; archaeal SC= 98.45%). Both were used to amplify gene region 3-5 and had mean lengths of 460 and 457, respectively. Thirty-three (OP_F114-KP_R031) or 34 (OP_F114-OP_R070) bacterial and three archaeal species from the oral cavity were not covered by these pairs. Lowering the cut-off level to SC ≥ 90.00% revealed six pairs with a longer mean sequence. For five of these, the difference was irrelevant (461 bps); but the pair OP_F114-OP_R073 had a mean length of 549. This combination targeted gene region 3-6 and had bacterial and archaeal SC values of 94.80 and 93.30%, respectively. The number of non-covered species increased to 40 for the bacteria and 13 for the archaea.

No primer pair from the >600 bps category had SC values ≥ 95.00% in either database. Conversely, 28 had bacterial and archaeal SC values ≥ 90.00% (bacterial SC range= 94.54–90.64%; archaeal SC range= 96.91–96.39%). These pairs amplified gene region 3-9, 4-9, or 5-9, had mean lengths between 622 and 1063 bps, and did not cover from 42 to 72 bacterial and six to seven archaeal species. The combination of OP_F066 with KP_R060, KP_R076, and OP_R121 yielded the highest coverage values, with all of them targeting region 5-9. Forty-two bacterial and six archaeal species were not covered by these primers. However, their mean sequence lengths were 623, 622, and 622 bps, respectively, which was close to the lower limit of this category. Primer pairs formed by OP_F114 with KP_R060, KP_R076, or OP_R121, which targeted region 3-9, had a better balance between the coverage results (bacteria SC= 91.42%, archaea SC= 96.91%) and the mean sequence lengths (1063, 1062, and 1062 bps). Sixty-six bacteria and six archaea were not covered by these pairs.

For each amplicon length category, we selected at least one primer pair suitable for detecting bacteria and archaea in the distinct 16S rRNA gene regions (Table 4). The pairs had to have SC ≥ 90.00% in both domains and were chosen based on their coverage and mean amplicon lengths. The VC results of these selected primers are detailed in the Additional file 14.

**Table 4:** Selected primer pairs for simultaneously detecting oral bacteria and archaea in different amplicon-length categories

		Bacteria					Archaea				
ALC (bps)	Primer pair	Gene region	SC (%)	Covered	Not covered	Mean length	Gene region	SC (%)	Covered	Not covered	Mean length
100–300	OP_F114-KP_R002	3-4	92.46	711	58	188	3	98.97	192	2	152
	KP_F020-KP_R032	4-5	95.58	735	34	284	3-5	99.48	193	1	285
	OP_F066-OP_R073	5-6	98.31	756	13	110	5	92.78	180	14	114
301–600	OP_F114-KP_R031	3-5	95.71	736	33	457	3-5	98.45	191	3	422
	OP_F114-OP_R073	3-6	94.80	729	40	549	3-5	93.30	181	13	518
	KP_F020-OP_R073	4-6	95.71	736	33	376	3-5	92.78	180	14	381
>600	OP_F114-OP_R121	3-9	91.42	703	66	1062	3-9	96.91	188	6	1035
	KP_F020-OP_R121	4-9	91.55	704	65	888	3-9	96.91	188	6	897
	OP_F066-OP_R121	5-9	94.54	727	42	622	5-9	96.91	188	6	630

Species coverage was estimated as the number of species with at least one match in an ASV divided by the number of species included in the database. Our bacterial and archaeal databases contained 769 and 194 species, respectively, each of which had between one and 4000 ASVs. The location of the first and last nucleotides of each primer within each sequence with a match was calculated and the mode values for these positions were determined. If there was more than one mode for a position, we chose the one closest to the mean position value. As all the sequences in the two databases were aligned with the 16S rRNA *E. coli* gene, the mode values obtained for each primer enabled us to allocate them to one of the gene regions defined for that organism by Baker et al. [55]. The reference sequence utilised had 1542 bps distributed in 10 conserved (C1–C10) and nine hypervariable regions (V1–V9). The sequences of these selected primer pairs are described in Additional file 15

*ALC* amplicon length category, *bps* base pairs, *F* forward, *KP* Klindworth primer, *OP* oral primer, *R* reverse, *SC* coverage at the species level

Additional file 18 is comprised of the species not covered by the pairs with a bacterial and an archaeal SC ≥ 95.00%, and by the combinations with a bacterial and archaeal SC ≥ 90.00% referred to in this section or included in Table 4.

Finally, Additional file 19 contains a list of the primer pairs used in the reviewed studies on 16S rRNA sequencing of the oral microbiome; and Additional file 20 details the species not covered by the three most frequently employed primer combinations in the literature.

## Discussion

To the best of our knowledge, this is the first study that evaluates in silico the coverage of 16S rRNA gene primers for the detection of oral-bacterial and archaeal species. The primer sequences were obtained not only from sequencing-based studies of the microbiota inhabiting the human mouth, but also from an article containing primers used in ecosystems as dissimilar as the marine, geothermal, human gut, or cattle gut [16]. Thus, numerous primers from diverse ecosystems were analysed to find those which performed better in the oral cavity. Moreover, to perform the analysis, we improved an earlier database of 16S rRNA gene sequences of oral bacteria [33] and created another from scratch that contained sequences from archaeal species found in the oral cavity.

We identified a series of individual primers that performed well in the detection of oral bacteria and/or archaea and combined them to create primer pairs. These were defined as “bacteria-specific,” “archaea-specific,” or “bacterial and archaeal” based on the results of their levels of coverage set out in the two databases. We also produced a series of primer pairs that may be the most suitable combinations for use when sequencing the oral ecosystem. These were classified according to the domain targeted, their mean amplicon length category, and the 16S rRNA gene region amplified.

### Comparative analysis of our coverage results of 16S rRNA gene primers with the literature

The investigation by Klindworth et al. [16] is perhaps the most comprehensive to date on the coverage and phylum spectrum of 16S rRNA primers. These authors assessed 175 primers and 512 primer pairs in silico against the Silva non-redundant reference database (version 108) [26], producing a selection of those that performed best for bacteria and archaea. Like us, this group organised the most suitable primer combinations for the different sequencing technologies into three categories according to their amplicon length (100–400, 400–1000, and >1000 bps). They then re-evaluated their analysis using the Global Ocean Sampling (GOS) dataset [56, 57], which is limited to the marine habitat, and they examined experimentally the primer pair that performed best [16].

We identified two investigations involving the oral ecosystem that used the Silva database [26]—versions 111 [19] or 132 [22]—to analyse the efficiency of 16S rRNA gene primers for detecting the archaea diversity in oral samples [22], or for reconstructing the microbiome of ancient dental calculus specimens [19]. Also, a third study evaluated the potential of seven primer pairs for detecting 219 species in a foregut dataset they created, which included oral, oesophageal, and gastric 16S rRNA gene sequences [23]. The pair with the best results for classifying the foregut genes was also analysed against the RDP database [27].

The numbers of 16S rRNA gene primer pairs evaluated in these three oral-related studies is substantially lower than in the present investigation: 12 individual primers combined to form 12 primer pairs [22]; 25 individual primers combined into 14 pairs [19]; and 14 individual primers grouped into 14 pairs [23]. In our study, we analysed 369 distinct individual primers and 4638 different primer-pair combinations. On the other hand, two investigations used the Silva database [19, 22, 26] which has broad phylogenetic diversity and contains information applicable to many environments but also includes 16S rRNA gene sequences that are misannotated taxonomically [33]. Specifically, comprehensive databases such as Silva [26], RDP [27], or Greengenes [58] have been estimated to have annotation error rates ranging from 17% to 10% [28], and their accuracy may also be reduced because they contain numerous sequences derived from some environments and only a few from others [29]. Furthermore, the evaluation of the primers’ coverage using an ecosystem-specific database, as in our study, would allow researchers to identify the species covered and not covered by a particular primer pair. In this sense, only Nossa et al. [23] evaluated the primer pairs against a self-created database containing sequences from their three niches of interest: oesophageal, oral, and gastric (together described as the foregut). Nevertheless, although this database contained 9484 sequences, only 2373 were oral and, overall, they represented just 219 bacterial species. These numbers are much lower than those in the bacteria database used in our study, which is based on eHOMD [34], and to which we added sequences from our self-created archaeal dataset (bacteria: 223,143 sequences, 769 species; archaea: 2842 sequences, 194 species).

In the present study, none of the individual primers yielded an SC= 100% when analysed against the oral bacteria or archaea databases. Due to this, it would not be possible to obtain a primer combination with such value as the coverage estimates of primer pairs are always lower than the values of the individual primers that form it. However, we do not know whether any of the primers included in this research could obtain such a value if mismatches were admitted.

#### Bacteria-specific primer pairs

Table 5 summarises our results and those in other publications, with the primer pairs ordered by the mean amplicon length and the domain targeted. Concerning the bacteria-specific candidates, our coverage estimates for KP_F047-KP_R021 (100–300 bps), KP_F049-KP_R033 (301–600 bps), KP_F056-KP_R074 (301–600 bps), KP_F033-KP_R060 (>600 bps), and KP_F047-KP_R053 (>600 bps) were similar to those of other studies, with differences no greater than 5.00% for both the bacteria and archaea domains [16]. It should be noted that the latter, classified here as having a mean amplicon length > 600 bps but put in the medium-length category by Klindworth [16], had a lower bacterial coverage when analysed against the GOS database [56, 57]. This was also the case for KP_F056-KP_R074 [16]. Moreover, the coverage values of the pairs KP_F077-KP_R071 (100–300 bps) and KP_F047-KP_R035 (301–600 bps) in other studies were similar to those in our research, but the archaeal coverage was notably higher—~31.00% [19] and ~83.00% [21, 25] more, respectively. KP_F047-KP_R035, which had an archaeal SC= 0.00% in our analysis, has been described elsewhere as having universal coverage for both archaea and bacteria [25]. We, therefore, believe that KP_F047-KP_R035 has value for detecting archaea in environmental [21, 25] or human gut [25] specimens, but not in samples from the oral cavity.

**Table 5:** Coverage findings described in the literature for the gene primer pairs analysed in the present study

Present study		Other studies	Results present study		Results other studies		Ref*.*
Primer pair	Mean length (bps)	Primer pair name	Bacterial SC (%)	Archaeal SC (%)	Bacterial coverage (%)	Archaeal coverage (%)	
KP_F044-KP_R023	100–300	S-D-Bact-0337-a-S-20/S-D-Bact-0518-a-A-17	87.52	0.00	80.90	0.00	[17]
KP_F044-KP_R021	100–300	S-D-Bact-0337-a-S-20/S-D-Bact-0515-a-A-19	92.46	0.00	85.80	0.00	[17]
KP_F046-KP_R023	100–300	S-D-Bact-0341-a-S-17/S-D-Bact-0518-a-A-17	87.52	0.00	81.30	0.00	[17]
KP_F046-KP_R021	100–300	S-D-Bact-0341-a-S-17/S-D-Bact-0515-a-A-19	92.46	0.00	86.20	0.00	[17]
KP_F046-OP_R045	100–300	S-D-Bact-0341-a-S-17/ N/A	87.52	0.00	81.50	0.00	[17]
KP_F047-KP_R021	100–300	S-D-Bact-0341-b-S-17/ S-D-Bact-0515-a-A-19	92.59	0.00	91.20^a^	0.00^a^	[16]
KP_F056-KP_R032	100–300	S-D-Bact-0564-a-S-15/S-D-Bact-0785-b-A-18	96.23	8.76	89.00^a^; 83.40^b^	14.60^a^; 0.00^b^	[16]
		S-D-Bact-0564-a-S-15/S-D-Bact-0785-b-A-18			88.10	14.40	[17]
KP_F058-KP_R053	100–300	S-D-Bact-0784-a-S-19/S-D-Bact-1061-a-A-17	84.40	0.00	78.60	0.00	[17]
KP_F077-KP_R071	100–300	U341F – 534R	95.58	59.79	98.00	~ 91.00	[19]
KP_F078-OP_R010	100–300	515F – 806 R (original)	95.32	62.89	86.80	52.90	[22]
		S-*-Univ-0515-a-S-19/N/A			86.10	52.00	[17]
KP_F078-KP_R037	100–300	S-*-Univ-0515-a-S-19/S-D-Bact-0787-a-A-20	87.39	0.52	77.10	0.00	[17]
KP_F018-KP_R002	100–300	S-D-Arch-0349-a-S-17/S-D-Arch-0519-a-A-16	0.00	95.88	0.00^a^; 0.00^b^	76.80^a^; 74.50^b^	[16]
KP_F020-KP_R032	100–300	S-D-Arch-0519-a-S-15/S-D-Bact-0785-b-A-18	95.58	99.48	89.10^a^; 83.40^b^	88.00^a^; 76.50^b^	[16]
		519F – 785R			88.80	88.90	[22]
KP_F020-KP_R035	100–300	S-D-Arch-0519-a-S-15/S-D-Bact-0785-a-A-21	95.45	98.97	87.10^a^	86.50^a^	[16]
OP_F014-OP_R014	100–300	515F – 806 R (modified)	95.32	88.14	87.70	85.70	[22]
		515F – 806R			96.20	96.39	[25]
OP_F066-OP_R073	100–300	N/A/ N/A	98.31	92.78	88.90	75.30	[17]
KP_F044-KP_R032	301–600	S-D-Bact-0337-a-S-20/S-D-Bact-0785-b-A-18	94.28	0.00	84.30	0.00	[17]
KP_F046-OP_R010	301–600	S-D-Bact-0341-a-S-17/ N/A	93.89	0.00	83.30	0.10	[17]
KP_F047-KP_R035	301–600	S-D-Bact-0341-b-S-17/S-*-D-Bact-0785-a-A-21	94.15	0.00	86.20^a^; 43.10^b^	0.50^a^; 0.00^b^	[16]
		341F -805R			96.69	83.59	[25]
		341F -785R			96.51	82.96	[21]
		S-D-Bact-0341-b-S-17/S-D-Bact-0785-a-A-21			86.00	0.50	[17]
KP_F049-KP_R033	301–600	S-D-Bact-0347-a-S-19/S-D-Bact-0785-a-A-19	76.59	0.00	76.50^a^	0.00^a^	[16]
KP_F056-KP_R074	301–600	S-D-Bact-0564-a-S-15/S-Univ-1100-a-A-15	97.27	7.73	92.70^a^; 76.20^b^	8.00^a^; 0.00^b^	[16]
OP_F021-OP_R050	301–600	N/A / N/A	91.68	1.03	86.50	0.50	[17]
KP_F020-KP_R013	301–600	S-D-Arch-0519-a-S-15/S-D-Arch-1041-a-A-18	0.00	95.88	0.00^a^	76.60^a^	[16]
KP_F032-KP_R063	>600	S-D-Bact-0008-b-S-20/S-D-Bact-1492-a-A-16	60.47	0.00	17.30	0.00	[17]
KP_F033-KP_R060	>600	S-D-Bact-0008-c-S-20/S-D-Bact-1391-a-A-17	74.90	0.00	78.00^a^	0.10^a^	[16]
KP_F033-KP_R050	>600	S-D-Bact-0008-c-S-20/S-D-Bact-1046-a-A-19	72.56	0.00	81.30^a^	0.00^a^	[16]
KP_F047-KP_R053	>600	S-D-Bact-0341-b-S-17/S-D-Bact-1061-a-A-17	93.11	0.00	91.90^a^; 58.90^b^	0.00^a^; 0.00^b^	[16]
KP_F051-KP_R057	>600	S-D-Bact-0515-a-S-16/S-D-Bact-1100-a-A-15	82.70	0.00	77.30	0.00	[17]
KP_F018-KP_R078	>600	S-D-Arch-0349-a-S-17/S-*-Univ-1392-a-A-15	0.00	93.30	0.00^a^	65.80^a^	[16]
KP_F059-KP_R078	>600	S-D-Bact-0785-a-S-18/S-*-Univ-1392-a-A-15	93.63	96.39	74.10^a^	72.30^a^	[16]

The coverage findings from the other investigations are those obtained when zero mismatches were accepted

*F* forward, *KP* Klindworth primer, *OP* oral primer, *R* reverse, *Ref* references, *SC* coverage at the species level

^a^Silva database, ^b^Global Ocean Sampling database

The remaining candidates to be bacteria-specific primer pairs in all the amplicon length categories herein were better at detecting bacteria than in other studies, with differences of 43.00% to 5.00%. Only Klindworth’s [16] estimate for the bacterial coverage of KP_F033-KP_R050 was better than ours, with an approximate difference of ~9.00% between the studies (Table 5).

#### Archaea-specific primer pairs

Two of the primer pairs selected herein to detect oral-archaea species—KP_F018-KP_R002 (100–300 bps) and KP_F020-KP_R013 (301–600 bps)—have previously been described in other studies as the best options for targeting this domain [16]. Nonetheless, the archaeal SC values we obtained exceed Klindworth’s [16] by ~ 20.00% (Table 5). Klindworth’s group [16] also found that KP_F018-KP_R078 had the highest overall archaeal coverage for the amplicons with a long mean length. However, they did not recommend its use due to its low phylum spectrum. This combination produced bacterial SC values of 0.00% and an archaeal SC of 93.30% when analysed against our database (Table 5). Although this is a good result, we prefer OP_F114-KP_R013, which achieves better archaeal SC, or KP_F018-KP_R063, which produces both better archaeal SC and a greater mean amplicon length.

#### Bacterial and archaeal primer pairs

KP_F020-KP_R032 and KP_F020-KP_R035 (100-300 bps), with bacterial and archaeal coverage estimates >85.00% (mainly considering the Silva database [26]), have been proposed previously as suitable for the detection of both domains [16]. As seen in Table 5, the SC values obtained herein, ≥95.00%, are better than those in other studies [16, 22]. OP_F066-OP_F073 is also among the favoured primers in our study for the detection of bacteria and archaea when using short amplicon lengths (SC= 98.31% and 92.78%, respectively), achieving better coverage than in the research by Zhang et al. [17] (SC= 88.90% and 75.30%, respectively). Meanwhile, although other studies’ in silico analyses of OP_F014-OP_R014 have described it as a good primer pair for detecting the two domains [22, 25], it is not among our recommended primers, since others in the same length and gene-region categories achieved better archaeal coverage. KP_F059-KP_R078 has been proposed by Klindworth et al. [16] as suitable for use with both the bacteria and archaea domains when employing medium mean amplicon lengths (608 bps). However, its length was 622 bps in our study, meaning that it was included in the >600 bps group. In any case, although our coverage values were higher than those obtained previously (SC= 93.63% and 96.39% *vs* 74.10% and 72.30%, respectively) (Table 5), other primer pairs performed better in both categories as OP_F114-KP_R031 (301–600 bps; bacterial SC= 95.71% and archaeal SC= 98.45%) and OP_F066-OP_R121 (>600 bps; bacterial SC= 94.54% and archaeal SC= 96.91%).

### Non-covered species by the 16S rRNA gene primer pairs

The in silico analysis has enabled us to verify that, among the pairs achieving better coverage, the species not covered by the primers targeting a particular region tend to be covered by others relating to a different zone. In this sense, most of the species that were not covered by the bacteria-specific primer pairs from regions 3-4 (100–300 bps), 3-5 (301–600 bps), or 3-7 (>600 bps) were by those from 5-7 and 6-7 (100–300 bps), 4-7 and 7-9 (301–600 bps), or 4-9 (>600 bps), and vice versa. This was also seen in the archaea-specific primers, where taxa not detected by the pairs from regions 3 (100–300 bps), 3-5 (301–600 bps), or 3-6 (>600 bps) were covered by those from 5-6 (100–300 bps), 3-6 and 5-9 (301–600 bps), or 3-9 and 5-9 (>600 bps), and vice versa. Lastly, the pairs for the two domains combined also demonstrated that species not covered by primers from zones 4-5 (100–300 bps) or 3-5 (301–600 bps) were by those from 5-6 (100–300 bps) or 4-6 (301–600 bps), and vice versa. In the combinations with mean amplicon lengths >600 bps, half the taxa that were not covered using primers for amplifying region 3-9 were detected when targeting 5-9. However, in this case, the opposite was not true.

There were exceptions to this general rule, which demonstrated that even for two primer pairs targeting the same gene region, one would be able to cover most of the species that were not detected by the other. As an example, the bacteria-specific pair KP_F048-OP_R043 detected 18 of the 33 species not covered by 18 different primer pairs formed by combining KP_F044, 046, 047, OP_F048, 096, or 108 and KP_R071, OP_R040, or 146 (gene region 3-4; 100-300 bps); and, also, OP_F101-OP_R030 covered 33 of the 62 species not detected by KP_F061-KP_R074 (6-7; 100-300 bps). Furthermore, 54 of the 75 bacteria not covered by KP_F048-OP_R050 were detected using KP_F048-OP_R073 and OP_F050-OP_R073 (3-6; 301-600 bps); and 22 of the 28 species not detected by KP_F051-KP_R053 and OP_F021-KP_R053 were covered by all the other primers targeting region 4-7 (301-600 bps). Also, in the long primer pairs, KP_F048-OP_R030 (3–7) covered 20 of the 37 non-detected taxa by the combinations of KP_F044, 046, 047, OP_F048, 096, or 108 with KP_R074. Meanwhile, the archaea-specific primer KP_F016-KP_R002 covered almost all (6/8) of the taxa not detected using KP_F018-KP_R002, -KP_R003, and -OP_R102 (3, 100-300 bps). In addition, KP_F016-KP_R032 was the only primer from region 3-5 (301-600 bps) able to identify six archaea: *Candidatus Korarchaeum cryptofilum*, *Ferroplasma acidarmanus*, *Fervidicoccus fontis*, *Metallosphaera cuprina*, *Methanocorpusculum labreanum*, and *Thermophilus pendens*; that were not detected by the rest of the primers from the same region. Nevertheless, these exceptions were not observed in the bacterial and archaeal primer pairs.

It is clear that most of the taxa not detected by these well-performing primer pairs must have been identified as present in the oral cavity at some point, or they would not have been included in the databases used for our in silico analysis. However, some of them are microbes associated with prevalent oral pathologies, such as periodontal disease or dental caries. We distinguished four recognised *Campylobacter* species among the bacteria not detected by some of the bacteria-specific primer pairs: *concisus*, *gracilis*, *rectus*, and *showae*. The first of these, as part of the Socransky green complex, has traditionally been associated with periodontal health; the remaining three are components of the orange complex, which is related to periodontitis [59]. A further three bacteria commonly found in the healthy periodontium, *Leptotrichia bucalis* [60], *Leptotrichia hofstadii* [60], and *Rothia dentocariosa* [61, 62], were also missed by some of the bacteria-specific and/or bacterial and archaeal primer pairs. Conversely, a few failed to cover bacterial taxa isolated from periodontally-diseased sites (in teeth or implants) or those regarded as novel periodontal pathogens, e.g., *Actinomyces dentalis* [63], *Actinomyces israelii* [63], *Desulfomicrobium orale* [64], *Mogibacterium timidum* [65], *Solobacterium moorei* [66, 67], *Treponema lecithinolyticum* [61, 65, 67], and *Treponema maltophilum* [68]. A further *Actinomyces* species, previously classified as *naeslundii* WVA 963 and now known as *johnsonii* [69], which has been encountered in both healthy and periodontitis sites [63], was not detected by some of the pairs that produced better coverage estimates. Moreover, different taxa from the phyla *Saccharibacteria* (TM7), which growing evidence links to periodontal disease [70], were also not covered. Meanwhile, the caries-associated bacterial species that were not detected by some of the primer pairs included *Bifidobacterium dentium* [71, 72], *Lactobacillus reuteri* [73], *Leptotrichia buccalis* [74], *Parascardovia denticolens* [73, 75], *R. dentocariosa* [76], and *Scardovia wiggsiae* [77].

The undetected archaeal species by some of the archaea-specific and/or bacterial and archaeal primer pairs included *Methanobrevibacter gottschalkii*, *Methanopyrus kandleri*, *Nitrosoarchaeum limnia*, and *Nitrososphaera evergladensis*; these species have been found, in order, in inflamed pulp tissue [78], periodontitis samples [79], endodontic infections [80], and ancient dental calculus [81, 82]. The rest of the non-detected archaea were extracted from the same publication [83] and, as far as we know, not reported by other authors so their role in the oral cavity has yet to be investigated.

Consequently, it would be preferable to choose a primer pair based on the health or disease condition being investigated. If it is known which oral species are not covered by each primer pair in the oral-specific database as we demonstrated for the first time in this study, and which taxa are most commonly associated with the target oral condition, it is possible to select the most optimal primer pair to use in the sequencing-based studies of the oral microbiota.

### Primer pairs frequently used in the oral microbiome literature

Finally, our review of the literature found that 206 distinct primer pairs have been utilised to study the oral microbiota via massive sequencing techniques. The combinations employed most commonly were KP_F078-OP_R010 and KP_F047-KP_R035, which were repeated 33 and 21 times, respectively. These were followed by KP_F014-KP_R011, KP_F034-KP_R065, KP_F031-KP_R021, and OP_F009-OP_R029, which appeared in eight, eight, seven, and seven articles, respectively. Four, three, four, 10, and 21 distinct pairs were repeated six, five, four, three, and two times in the sequencing-based studies of the oral microbiome. Lastly, 158 were found only once.

Only 67 of these 206 pairs were evaluated in the present study. This means that at least one of the individual primers from the remaining 139 combinations had a bacterial and archaeal SC < 75.00%. The widely employed primer KP_F078-OP_R010, which targets region 4 and is typically found as 515F-806R, was developed by Caporaso et al. [84] for use in the Illumina sequencing platform. The in silico analysis herein revealed bacterial and archaeal SC estimates of 95.32% and 62.89%, respectively (mean amplicon length= 292 bps), but failed to detect *M. kandleri*, *N. limnia*, and *N. evergladensis*, among other archaeal species. Numerous primer combinations in the same length category (100–300 bps) and targeting the same gene region (4-5) provided better SC for both domains, e.g., KP_F020 and KP_R031, KP_R032, or OP_R070 (bacterial SC range= 95.97–95.58%; archaeal SC range= 99.48–98.97%; mean amplicon length range= 287–284 bps). If only bacteria are to be detected, the primer pair from the same region, OP_F098-OP_R119, is preferrable; although it had a slightly lower bacterial SC (94.54%), its archaeal SC was 0.00%, meaning that no 16S rRNA gene sequence from an oral archaeon would limit the sequencing depth.

KP_F047-KP_R035, directed to amplify region 3-4, has been referred to as 341F-785R, 341F-805R, or 341F-806R and is the pair proposed in the Illumina protocol for the preparation of the sequencing library [85]. In the in silico analysis, it achieved species coverages of 94.15% and 0.00% in the oral-bacteria and oral-archaea databases, respectively, (mean amplicon length= 460 bps). Surprisingly, this pair did not cover the previously mentioned bacterial species *A. dentalis*, *A. israelii*, *A. johnsonii*, *D. orale*, *L. reuteri*, *M. timidum*, *T. lecithinolyticum*, and *T. maltophilum*. Although it has been used extensively in oral microbiome studies, in our investigation other primers in the same length category (301–600 bps) and the same gene region (3–5) had better bacterial coverage values and a similar mean amplicon length as KP_F048-KP_R031 (SC= 97.53%). This latter pairing, as well as all those in the same length category and targeting the same region, also failed to detect *A. dentalis*, *A. israelii*, *A. johnsonii* and *D. orale.* However, unlike KP_F047-KP_R035, *L. reuteri*, *M. timidum*, *T. lecithinolyticum*, and *T. maltophilum* were covered.

Another widely used primer is 785F-1175R, which has been employed to amplify gene region 5-7. The in silico evaluation of the pair named herein as OP_F009-OP_R029 yielded bacterial SC values of 88.30% and an archaeal SC of 0.00% (mean amplicon length= 410 bps). This, along with all the other bacteria-specific combinations within the same gene region (5–8) and amplicon length category (301–600 bps), was not among the best primers in our investigation (bacterial SC range= 89.60–79.97%; archaeal SC= 0.00%; mean amplicon length range= 411–408 bps). In fact, OP_F009-OP_R029 not only failed to detect *A. dentalis*, *A. israelii*, *A. johnsonii, C. concisus, C. gracilis, C. rectus*, and *C. showae*, but also the microbes that are widely known to be associated with periodontitis, *Porphyromonas endodontalis* [86–88], *Porphyromonas gingivalis* [59, 86, 88, 89], and *Tannerella forsythia* [59, 88, 89]. Consequently, it is preferable to amplify region 4-7 using the pairs KP_F051-OP_R030 or OP_F021-OP_R030, which had better bacterial SC (98.83% and 98.70%, respectively) and mean amplicon lengths (566 bps), and also detected the bacterial species referred to above.

### Factors to consider when selecting a 16S rRNA gene primer pair

Although we defined which primer pairs had the highest coverage results for detecting oral bacteria and archaea, this does not necessarily mean that they would be always the best option for any sequencing-based research on the oral microbiome. Other factors such as the amplicon length or gene region targeted should also be taken into account when selecting the optimum primer pair as we did for constructing Tables 2, 3, and 4. Although PCR efficiency decreases when the amplicon length increases [90], in general terms, the longer the fragment sequenced, the lower the taxonomic level that can be achieved [17]. Indeed, sequencing full-lengths, as is possible with PacBio, is regarded as the solution to the limitations of taxonomic classification [17]. Nevertheless, Soergel et al. [29] evaluated primer pairs in common use and found that longer gene amplicons did not necessarily confer better classifications, with the target region (depending on the sample’s origin) impacting taxonomic assignment the most. Similarly, other authors have recently found that the different 16S rRNA gene regions contain varying amounts of information, which significantly affects the composition of the bacterial community [17]. Consequently, we agree that the choice of the target region is also an important factor [17, 29].

In this sense, our study provides the scientific community with information on these three aspects to consider in the selection of a primer pair for a total of 4638 primer pairs, adding the description of the covered and non-covered taxa for each primer pair. Our goal is to enable researchers to select the best primer pair to meet their research expectations.

In view of our results, the bacteria-specific primer pairs showed very similar average coverage values in the three mean amplicon length categories (100-300 bps: 94.19%; 301-600 bps: 94.71%; >600 bps: 94.87%). However, the archaea-specific primer combinations with short mean amplicon lengths had a slightly higher overall coverage than those from the other two categories (100-300: 95.54% *vs.* 301-600: 93.30% and >600: 93.81%); and the bacterial and archaeal primer pairs with short and long mean amplicon lengths performed better than those from the medium category (100-300: 97.08% and >600: 96.91% *vs.* 301-600: 94.83%). Given the above, as the differences in overall coverage between the three amplicon length categories were not too large, the choice of amplicon length, and consequently the sequencing platform, is left to the discretion of the researchers. On the other hand, the gene regions showing the greatest coverage values in the three mean amplicon length categories (in order 100-300, 301-600, and >600) were as follows: 3-4, 4-7, and 3-7 (bacteria-specific); 3 or 5-6, 3-5 or 3-6, and 3-6 (archaea-specific); and 4-5, 3-5, and 5-9 (bacterial and archaeal). Thus, for the different lengths of the bacteria-specific primers and the bacterial and archaeal primers, there was a specific region associated with the highest coverage, which was not observed in the archaea-specific primers where greater variability was detected. Moreover, except for the archaeal-specific and the bacterial and archaeal primer pairs in the medium mean amplicon length category, in which region 3-5 showed the highest coverage estimates; there was no consensus between the three types of primer pairs on the most informative region for a given category.

### Limitations of the present study

The main limitation of the present study arises from the lack of information on the first and last positions in the sequence annotations stored by the NCBI [37], which suggests that primers targeting these gene regions may have lower VC values. We, therefore, calculated the SC estimates, since a particular species would be regarded as covered by a particular primer if at least one of its variants is amplified. In addition, we were unable to identify the complete genome of some archaeal species in our database (*C. K. cryptofilum*, *M. gottschalkii strain HO*, *Methanobrevibacter oralis*, *Methanobrevibacter thaueri strain CW*, and *N. limnia*). Given that the gene sequences from these taxa were not obtained in the same way as for the other species, we cannot be sure that there are no sequence variants in addition to those found in our investigation. It should, however, be noted that the oral archaea database developed by our group is the first proposal and may, therefore, be subject to change. Moreover, additional scientific evidence on the archaea species associated with the oral cavity and its diseases is required to increase the amount of information contained in the 16S rRNA sequence databases.

In consequence, the results of our in silico analysis have potential use in studies of the oral microbiome and need to be confirmed in other experimental studies using omics techniques.

## Conclusions

Considering the three amplicon category lengths (100–300, 301–600, and >600 bps), the primer pairs with the best-estimated coverage for detecting oral bacteria targeted regions 3-4, 4-7, and 3-7 were as follows: KP_F048-OP_R043 (primer pair position for *Escherichia coli* J01859.1: 342-529), KP_F051-OP_R030 (514-1079), and KP_F048-OP_R030 (342-1079). For the detection of oral archaea, the pairs with the best coverage amplified regions 5-6, 3-6, and 3-6 were as follows: OP_F066-KP_R013 (784-undefined), KP_F020-KP_R013 (518-undefined), and OP_F114-KP_R013 (340-undefined). The pairs with the best coverage of the bacteria and archaea domains jointly were found in regions 4-5, 3-5, and 5-9, and these were as follows: KP_F020-KP_R032 (518-801), OP_F114-KP_R031 (340-801), and OP_F066-OP_R121 (784-1405). The primer pairs with the best coverage identified herein are not among those described most widely in the oral microbiome literature.

## Supplementary Information

**Additional file 1: **List of words employed in the automated searches to identify the 16S rRNA gene primers used for detecting oral bacteria and oral archaea before sequencing, and to elaborate a list of oral-archaea species.

**Additional file 2: **List of references from which a particular primer was initially obtained.

**Additional file 3: **List of references from which the archaeal species inhabiting different human-mouth niches were obtained.

**Additional file 4: **Forward and reverse 16S rRNA gene primers that were evaluated in the study and the sequence comparison which was used to detect repeats.

**Additional file 5: **List of archaeal species present in the human mouth and the PMID of the investigations from which they were obtained.

**Additional file 6: **Oral-bacteria database of the 16S rRNA gene sequences which was used in the present study for the coverage analysis.

**Additional file 7: **16S rRNA gene sequences from the oral archaea which were employed for the BLASTN search against the NCBI non-redundant nucleotide database.

**Additional file 8: **Oral-archaea database of the 16S rRNA gene sequences constructed by our group before alignment.

**Additional file 9: **Oral-archaea database of the 16S rRNA gene sequences which was used in the present study for the coverage analysis.

**Additional file 10: **Information related to the coverage analysis of the 16S rRNA gene individual primers.

**Additional file 11: **Evaluation of individual primers against the oral-bacteria database.

**Additional file 12: **Evaluation of individual primers against the oral-archaea database.

**Additional file 13: **Evaluation of primer pairs against the oral-bacteria and the oral-archaea databases.

**Additional file 14: **Coverage at the variant level of the selected primer pairs for detecting oral bacteria or/and archaea.

**Additional file 15: **Sequences of the selected primer pairs for detecting oral bacteria or/and archaea.

**Additional file 16: **Bacterial species non covered by the selected bacteria-specific primer pairs.

**Additional file 17: **Archaeal species non covered by the selected archaea-specific primer pairs.

**Additional file 18: **Species non covered by the selected primer pairs for detecting both bacteria and archaea.

**Additional file 19: **List of the primer pairs utilised in the reviewed oral microbiome studies through 16S rRNA gene sequencing.

**Additional file 20: **Oral-bacteria species non covered by KP_F078-OP_R010, KP_F047-KP_R035, and OP_F009-OP_R029; and oral-archaea species non covered by KP_F078-OP_R010.

## Data Availability

Principal data generated or analysed during this study are included in this published article.

## Abbreviations

ASV(s): Amplicon sequence variant(s)
bp(s): Base pair(s)
eHOMD: extended Human Oral Microbiome Database
F: Forward
GOS: Global Ocean Sampling
GTDB: Genome Taxonomy Database
KP: Klindworth primer
Max.: Maximum
Min.: Minimum
NCBI: National Centre for Biotechnology Information
NGS: Next-generation sequencing
OP: Oral primer
PacBio: Pacific Biosciences
PCR: Polymerase chain reaction
R: Reverse
RDP: Ribosomal Database Project
rRNA: Ribosomal RNA
SC: Coverage at the species level
UI: Unidentified
VC: Coverage at the variant level
WGS: Whole-genome shotgun
