# Supplementary material for: In silico evaluation and selection of the best 16S rRNA gene primers for use in next-generation sequencing to detect oral bacteria and archaea
Source: Microbiome. 2023 Mar 23;11:58. doi: 10.1186/s40168-023-01481-6 (PMC10035280; doi:10.1186/s40168-023-01481-6)
Supplement: Supplementary file 2 — Additional file 1: List of words employed in the automated searches to identify the 16S rRNA gene primers used for detecting oral bacteria and oral archaea before sequencing, and to elaborate a list of oral-archaea species. [file 40168_2023_1481_MOESM1_ESM.docx]

Additional table 1. Words employed to find 16S rRNA gene primers used for detecting oral bacteria before sequencing.

| **field1** | **field2** | **field3** | **field4** |
| --- | --- | --- | --- |
| oral | microbiome | sequencing | region |
| mouth | microbiota | "sanger sequencing" | "hypervariable region" |
| caries | bacteria | "454 sequencing" | 16S |
| "periodontal disease*" | microbe | "454 pyrosequencing" |  |
| periodontitis | "bacterial communit*" | Illumina |  |
| gingivitis | "microbial communit*" | "ion torrent" |  |
| oral cancer | "bacterial structure" | PacBio |  |
| supragingival | "microbial structure" |  |  |
| subgingival | "bacterial ecology" |  |  |
| saliva | "microbial ecology" |  |  |
| tongue |  |  |  |
| cheek |  |  |  |
| "buccal mucosa" |  |  |  |
| "oral mucosa" |  |  |  |

Each search included one word from each of the four fields. Field one contains 14 words related to the oral cavity and its diseases, field two is constituted by 10 terms associated with the microbiome and the bacterial diversity, field three has seven words related to several types of sequencing platforms and, finally, field four contains three terms associated with the 16S rRNA gene. All possible combinations between fields were performed, making a total of 2940 automated searches.

Additional table 2. Words employed to find 16S rRNA gene primers used for detecting oral archaea before sequencing, and to elaborate a list of oral-archaea species.

| **field1** | **field2** | **field3** | **field4** |
| --- | --- | --- | --- |
| oral | microbiome | sequencing | region |
| mouth | microbiota | "sanger sequencing" | "hypervariable region" |
| caries | microbe | "454 sequencing" | 16S |
| "periodontal disease*" | "microbial communit*" | "454 pyrosequencing" |  |
| periodontitis | "microbial structure" | Illumina |  |
| gingivitis | "microbial ecology" | "ion torrent" |  |
| oral cancer | archaeome | PacBio |  |
| supragingival | "archaeal communit*" |  |  |
| subgingival | "archaeal structure" |  |  |
| saliva* | "archaeal ecology" |  |  |
| tongue | archaea |  |  |
| cheek* | archaeal |  |  |
| "buccal mucosa" |  |  |  |
| "oral mucosa" |  |  |  |
| dental |  |  |  |
| pulp |  |  |  |
| peri-implantitis |  |  |  |
| endodontic* |  |  |  |
| periodontal |  |  |  |
| "root canal" |  |  |  |
| tooth |  |  |  |
| teeth |  |  |  |
| gingiva |  |  |  |

Again, each search included one word from each of the four fields. Field one contains 23 words related to the oral cavity and its diseases, field two is constituted by 12 terms associated with the microbiome and the archaeal diversity, field three has seven words related to several types of sequencing platforms and, lastly, field four contains three terms associated with the 16S rRNA gene. All possible combinations between the four fields were performed, meaning a total of 5796 automated searches.

To elaborate the list of archaeal species inhabiting the human mouth we used the fields one and two from additional table 2. The combination of these terms implied a total of 276 automated searches.
