# Supplementary material for: In silico evaluation and selection of the best 16S rRNA gene primers for use in next-generation sequencing to detect oral bacteria and archaea
Source: Microbiome. 2023 Mar 23;11:58. doi: 10.1186/s40168-023-01481-6 (PMC10035280; doi:10.1186/s40168-023-01481-6)
Supplement: Supplementary file 3 — Additional file 2: List of references from which a particular primer was initially obtained. [file 40168_2023_1481_MOESM2_ESM.docx]

Additional table 3. References from which we obtained at least one different 16S rRNA gene primer.

| **PMID** | **Reference** |
| --- | --- |
| 22933715 | (1) |
| 29138298 | (2) |
| 15067114 | (3) |
| 24859768 | (4) |
| 31739075 | (5) |
| 11267768 | (6) |
| 33024215 | (7) |
| 27135839 | (8) |
| 22251411 | (9) |
| 21539593 | (10) |
| 22561061 | (11) |
| 19572899 | (12) |
| 31866971 | (13) |
| 25797107 | (14) |
| 25830311 | (15) |
| 27010812 | (16) |
| 26461079 | (17) |
| 24671083 | (18) |
| 28871594 | (19) |
| 27262209 | (20) |
| 25398868 | (21) |
| 21738596 | (22) |
| 23489056 | (23) |
| 23762384 | (24) |
| 28808698 | (25) |
| 22170420 | (26) |
| 22675498 | (27) |
| 29668720 | (28) |
| 28766745 | (29) |
| 27680288 | (30) |
| 25946126 | (31) |
| 28780383 | (32) |
| 23967169 | (33) |
| 28886168 | (34) |
| 20920195 | (35) |
| 31281801 | (36) |
| 18946007 | (37) |
| 30397210 | (38) |
| 29576948 | (39) |
| 14576113 | (40) |
| 32541791 | (41) |
| 17600539 | (42) |
| 21853142 | (43) |
| 25683180 | (44) |
| 30185233 | (45) |
| 26103050 | (46) |
| 26984526 | (47) |
| 26563586 | (48) |
| 11923328 | (49) |
| 22760039 | (50) |
| 26686411 | (51) |
| 19796657 | (52) |
| 24974898 | (53) |
| 23845046 | (54) |
| 22319750 | (55) |
| 32040467 | (56) |
| 19543549 | (57) |
| 2469776 | (58) |
| 27330858 | (59) |
| 16597879 | (60) |
| 12631201 | (61) |
| 18033835 | (62) |
| 17957246 | (63) |
| 28404012 | (64) |
| 20107092 | (65) |
| 20861633 | (66) |
| 30642327 | (67) |
| 29735410 | (68) |
| 18218926 | (69) |
| 29609564 | (70) |
| 31211815 | (71) |
| 27371581 | (72) |
| 25183372 | (73) |
| 16011760 | (74) |
| 31502122 | (75) |
| 19447073 | (76) |
| 16455901 | (77) |
| 16238598 | (78) |
| 12324358 | (79) |
| 27357925 | (80) |
| 22753064 | (81) |
| 31305026 | (82) |
| 31151886 | (83) |
| 28819242 | (84) |
| 16000454 | (85) |
| 31424011 | (86) |
| 11594611 | (87) |
| 19807788 | (88) |
| 22693635 | (89) |
| 22413030 | (90) |
| 25227214 | (91) |
| 25166027 | (92) |
| 26856712 | (93) |
| 30508628 | (94) |
| 22516132 | (95) |
| 19406777 | (96) |
| 27063111 | (97) |
| 28273061 | (98) |
| 25283067 | (99) |
| 23303375 | (100) |
| 29765908 | (101) |
| 23613868 | (102) |
| 21538072 | (103) |
| 31681625 | (104) |
| 25761675 | (105) |
| 24800728 | (106) |
| 30594172 | (107) |
| 27495902 | (108) |
| 24965363 | (109) |
| 31951641 | (110) |
| 21110215 | (111) |
| 22685668 | (112) |
| 22613548 | (113) |
| 28283005 | (114) |
| 27107811 | (115) |
| 16390343 | (116) |
| 20654735 | (117) |
| 30580031 | (118) |
| 24450871 | (119) |
| 21205002 | (120) |
| 25942317 | (121) |
| 27177110 | (122) |
| 25097398 | (123) |
| 29362114 | (124) |
| 27629863 | (125) |
| 20510651 | (126) |
| 24169577 | (127) |
| 27036492 | (128) |
| 28259623 | (129) |
| 28486933 | (130) |
| 24439218 | (131) |
| 31806002 | (132) |
| 8782684 | (133) |
| 11411720 | (134) |
| 31745056 | (135) |
| 20656903 | (136) |
| 19346352 | (137) |
| 17172525 | (138) |
| 32515429 | (139) |
| 20835612 | (140) |
| 7981097 | (141) |
| 25495549 | (142) |
| 2312676 | (143) |
| 11491323 | (144) |
| 15815015 | (145) |

**REFERENCES**

(1) Klindworth A, Pruesse E, Schweer T, Peplies J, Quast C, Horn M, et al. Evaluation of general 16S ribosomal RNA gene PCR primers for classical and next-generation sequencing-based diversity studies. Nucleic Acids Res. 2013;41:e1.

(2) Koskinen K, Pausan MR, Perras AK, Beck M, Bang C, Mora M, et al. First insights into the diverse human archaeome: specific detection of archaea in the gastrointestinal tract, lung, and nose and on skin. mBio. 2017;8:e00824-17.

(3) Lepp PW, Brinig MM, Ouverney CC, Palm K, Armitage GC, Relman DA. Methanogenic archaea and human periodontal disease. Proc Natl Acad Sci U S A. 2004;101:6176-81.

(4) Li CL, Jiang YT, Liu DL, Qian J, Liang JP, Shu R. Prevalence and quantification of the uncommon archaea phylotype thermoplasmata in chronic periodontitis. Arch Oral Biol. 2014;59:822-8.

(5) Dame-Teixeira N, de Cena JA, Côrtes DA, Belmok A, Dos Anjos Borges, LG, Marconatto L, et al. Presence of archaea in dental caries biofilms. Arch Oral Biol. 2020;110:104606.

(6) Kulik EM, Sandmeier H, Hinni K, Meyer J. Identification of archaeal rDNA from subgingival dental plaque by PCR amplification and sequence analysis. FEMS Microbiol Lett. 2001;196:129-33.

(7) Kumpitsch C, Moissl-Eichinger C, Pock J, Thurnher D, Wolf A. Preliminary insights into the impact of primary radiochemotherapy on the salivary microbiome in head and neck squamous cell carcinoma. Sci Rep. 2020;10:16582.

(8) Schuerger AC, Nicholson WL. Twenty-three species of hypobarophilic bacteria recovered from diverse ecosystems exhibit growth under simulated martian conditions at 0.7 kPa. Astrobiology. 2016;16:335-47.

(9) Ozok AR, Persoon IF, Huse SM, Keijser BJ, Wesselink PR, Crielaard W, et al. Ecology of the microbiome of the infected root canal system: a comparison between apical and coronal root segments. Int Endod J. 2012;45:530-41.

(10) Matarazzo F, Ribeiro AC, Feres M, Faveri M, Mayer MP. Diversity and quantitative analysis of archaea in aggressive periodontitis and periodontally healthy subjects. J Clin Periodontol. 2011;38:621-7.

(11) Horz HP, Seyfarth I, Conrads G. McrA and 16S rRNA gene analysis suggests a novel lineage of archaea phylogenetically affiliated with thermoplasmatales in human subgingival plaque. Anaerobe 2012 Jun;18(3):373-7.

(12) Li CL, Liu DL, Jiang YT, Zhou YB, Zhang MZ, Jiang W, et al. Prevalence and molecular diversity of archaea in subgingival pockets of periodontitis patients. Oral Microbiol Immunol 2009 Aug;24(4):343-6.

(13) Pausan MR, Csorba C, Singer G, Till H, Schöpf V, Santigli E, et al. Exploring the archaeome: detection of archaeal signatures in the human body. Front Microbiol 2019 Dec 5;10:2796.

(14) Horz HP, Robertz N, Vianna ME, Henne K, Conrads G. Relationship between methanogenic archaea and subgingival microbial complexes in human periodontitis. Anaerobe. 2015;35:10-2.

(15) Huynh HT, Pignoly M, Nkamga VD, Drancourt M, Aboudharam G. The repertoire of archaea cultivated from severe periodontitis. PLoS One. 2015;10:e0121565.

(16) Khelaifia S, Lagier JC, Nkamga VD, Guilhot E, Drancourt M, Raoult D. Aerobic culture of methanogenic archaea without an external source of hydrogen. Eur J Clin Microbiol Infect Dis. 2016;35:985-91.

(17) Camelo-Castillo A, Novoa L, Balsa-Castro C, Blanco J, Mira A, Tomás I. Relationship between periodontitis-associated subgingival microbiota and clinical inflammation by 16S pyrosequencing. J Clin Periodontol. 2015;42:1074-82.

(18) Li Y, He J, He Z, Zhou Y, Yuan M, Xu X, et al. Phylogenetic and functional gene structure shifts of the oral microbiomes in periodontitis patients. ISME J. 2014;8:1879-91.

(19) Pérez-Chaparro PJ, McCulloch JA, Mamizuka EM, Moraes, A D C L, Faveri M, Figueiredo LC, et al. Do different probing depths exhibit striking differences in microbial profiles? J Clin Periodontol. 2018;45:26-37.

(20) Tsai CY, Tang CY, Tan TS, Chen KH, Liao KH, Liou ML. Subgingival microbiota in individuals with severe chronic periodontitis. J Microbiol Immunol Infect. 2018;51:226-34.

(21) Kirst ME, Li EC, Alfant B, Chi YY, Walker C, Magnusson I, et al. Dysbiosis and alterations in predicted functions of the subgingival microbiome in chronic periodontitis. Appl Environ Microbiol. 2015;81:783-93.

(22) Kumar PS, Brooker MR, Dowd SE, Camerlengo T. Target region selection is a critical determinant of community fingerprints generated by 16S pyrosequencing. PLoS One. 2011;6:e20956.

(23) Bizzarro S, Loos BG, Laine ML, Crielaard W, Zaura E. Subgingival microbiome in smokers and non-smokers in periodontitis: an exploratory study using traditional targeted techniques and a next-generation sequencing. J Clin Periodontol. 2013;40:483-92.

(24) Ge X, Rodriguez R, Trinh M, Gunsolley J, Xu P. Oral microbiome of deep and shallow dental pockets in chronic periodontitis. PLoS One. 2013;8:e65520.

(25) Deng K, Ouyang XY, Chu Y, Zhang Q. Subgingival microbiome of gingivitis in chinese undergraduates. Chin J Dent Res. 2017;20:145-52.

(26) Griffen AL, Beall CJ, Campbell JH, Firestone ND, Kumar PS, Yang ZK, et al. Distinct and complex bacterial profiles in human periodontitis and health revealed by 16S pyrosequencing. ISME J. 2012;6:1176-85.

(27) Liu B, Faller LL, Klitgord N, Mazumdar V, Ghodsi M, Sommer DD, et al. Deep sequencing of the oral microbiome reveals signatures of periodontal disease. PLoS One. 2012;7:e37919.

(28) Hagenfeld D, Koch R, Junemann S, Prior K, Harks I, Eickholz P, et al. Do we treat our patients or rather periodontal microbes with adjunctive antibiotics in periodontal therapy? A 16S rDNA microbial community analysis. PLoS One. 2018;13:e0195534.

(29) Sanz-Martin I, Doolittle-Hall J, Teles RP, Patel M, Belibasakis GN, Hammerle CHF, et al. Exploring the microbiome of healthy and diseased peri-implant sites using Illumina sequencing. J Clin Periodontol. 2017;44:1274-84.

(30) Teng F, He T, Huang S, Bo CP, Li Z, Chang JL, et al. Cetylpyridinium chloride mouth rinses alleviate experimental gingivitis by inhibiting dental plaque maturation. Int J Oral Sci. 2016;8:182-90.

(31) Romani Vestman N, Chen T, Lif Holgerson P, Ohman C, Johansson I. Oral microbiota shift after 12-week supplementation with *lactobacillus reuteri* DSM 17938 and PTA 5289; a randomized control trial. PLoS One. 2015;10:e0125812.

(32) Apatzidou D, Lappin DF, Hamilton G, Papadopoulos CA, Konstantinidis A, Riggio MP. Microbiome of peri-implantitis affected and healthy dental sites in patients with a history of chronic periodontitis. Arch Oral Biol. 2017;83:145-52.

(33) Kistler JO, Booth V, Bradshaw DJ, Wade WG. Bacterial community development in experimental gingivitis. PLoS One. 2013;8:e71227.

(34) Sarkar A, Stoneking M, Nandineni MR. Unraveling the human salivary microbiome diversity in Indian populations. PLoS One. 2017;12:e0184515.

(35) Lazarevic V, Whiteson K, Hernandez D, Francois P, Schrenzel J. Study of inter- and intra-individual variations in the salivary microbiota. BMC Genomics. 2010;11:523.

(36) Lundmark A, Hu YOO, Huss M, Johannsen G, Andersson AF, Yucel-Lindberg T. Identification of salivary microbiota and its association with host inflammatory mediators in periodontitis. Front Cell Infect Microbiol. 2019;9:216.

(37) Keijser BJ, Zaura E, Huse SM, van der Vossen, J. M., Schuren FH, Montijn RC, et al. Pyrosequencing analysis of the oral microflora of healthy adults. J Dent Res. 2008;87:1016-20.

(38) Teng F, Darveekaran Nair SS, Zhu P, Li S, Huang S, Li X, et al. Impact of DNA extraction method and targeted 16S-rRNA hypervariable region on oral microbiota profiling. Sci Rep. 2018;8:16321.

(39) Wu Y, Chi X, Zhang Q, Chen F, Deng X. Characterization of the salivary microbiome in people with obesity. PeerJ. 2018;6:e4458.

(40) McBain AJ, Bartolo RG, Catrenich CE, Charbonneau D, Ledder RG, Gilbert P. Effects of triclosan-containing rinse on the dynamics and antimicrobial susceptibility of *in vitro* plaque ecosystems. Antimicrob Agents Chemother. 2003;47:3531-8.

(41) Lif Holgerson P, Esberg A, Sjödin A, West CE, Johansson I. A longitudinal study of the development of the saliva microbiome in infants 2 days to 5 years compared to the microbiome in adolescents. Sci Rep. 2020;10:9629.

(42) Siqueira JF,Jr, Rôças IN, Paiva SS, Magalhães KM, Guimarães-Pinto T. Cultivable bacteria in infected root canals as identified by 16S rRNA gene sequencing. Oral Microbiol Immunol. 2007;22:266-71.

(43) Cephas KD, Kim J, Mathai RA, Barry KA, Dowd SE, Meline BS, et al. Comparative analysis of salivary bacterial microbiome diversity in edentulous infants and their mothers or primary care givers using pyrosequencing. PLoS One. 2011;6:e23503.

(44) Macovei L, McCafferty J, Chen T, Teles F, Hasturk H, Paster BJ, et al. The hidden 'mycobacteriome' of the human healthy oral cavity and upper respiratory tract. J Oral Microbiol. 2015;7:26094.

(45) Mukherjee C, Beall CJ, Griffen AL, Leys EJ. High-resolution ISR amplicon sequencing reveals personalized oral microbiome. Microbiome. 2018;6:153.

(46) Chen J, Miao X, Xu M, He J, Xie Y, Wu X, et al. Intra-genomic heterogeneity in 16S rRNA genes in strictly anaerobic clinical isolates from periodontal abscesses. PLoS One. 2015;10:e0130265.

(47) Zhang Y, Ji P, Wang J, Zhao F. RiboFR-Seq: a novel approach to linking 16S rRNA amplicon profiles to metagenomes. Nucleic Acids Res. 2016;44:e99.

(48) Ziesemer KA, Mann AE, Sankaranarayanan K, Schroeder H, Ozga AT, Brandt BW, et al. Intrinsic challenges in ancient microbiome reconstruction using 16S rRNA gene amplification. Sci Rep. 2015;5:16498.

(49) Kaplan JB, Schreiner HC, Furgang D, Fine DH. Population structure and genetic diversity of *actinobacillus actinomycetemcomitans* strains isolated from localized juvenile periodontitis patients. J Clin Microbiol. 2002;40:1181-7.

(50) Zbinden A, Mueller NJ, Tarr PE, Eich G, Schulthess B, Bahlmann AS, et al. *Streptococcus tigurinus*, a novel member of the *streptococcus mitis* group, causes invasive infections. J Clin Microbiol. 2012;50:2969-73.

(51) Yu XL, Chan Y, Zhuang LF, Lai HC, Lang NP, Lacap-Bugler DC, et al. Distributions of Synergistetes in clinically-healthy and diseased periodontal and peri-implant niches. Microb Pathog. 2016;94:90-103.

(52) Lazarevic V, Whiteson K, Huse S, Hernandez D, Farinelli L, Osterås M, et al. Metagenomic study of the oral microbiota by Illumina high-throughput sequencing. J Microbiol Methods. 2009;79:266-71.

(53) Dhotre SV, Mehetre GT, Dharne MS, Suryawanshi NM, Nagoba BS. Isolation of *streptococcus tigurinus* - a novel member of *streptococcus mitis* group from a case of periodontitis. FEMS Microbiol Lett. 2014;357:131-5.

(54) da Silva ES, Feres M, Figueiredo LC, Shibli JA, Ramiro FS, Faveri M. Microbiological diversity of peri-implantitis biofilm by Sanger sequencing. Clin Oral Implants Res. 2014;25:1192-9.

(55) Chen Z, Trivedi HM, Chhun N, Barnes VM, Saxena D, Xu T, et al. Using DGGE and 16S rRNA gene sequence analysis to evaluate changes in oral bacterial composition. Chin J Dent Res. 2011;14:95-103.

(56) Kazemtabrizi A, Haddadi A, Shavandi M, Harzandi N. Metagenomic investigation of bacteria associated with dental lesions: a cross-sectional study. Med Oral Patol Oral Cir Bucal. 2020;25:e240-51.

(57) Gu F, Li Y, Zhou C, Wong DT, Ho CM, Qi F, et al. Bacterial 16S rRNA/rDNA profiling in the liquid phase of human saliva. Open Dent J. 2009;3:80-4.

(58) Chuba PJ, Pelz K, Krekeler G, de Isele TS, Göbel U. Synthetic oligodeoxynucleotide probes for the rapid detection of bacteria associated with human periodontitis. J Gen Microbiol. 1988;134:1931-8.

(59) Bisanz JE, Suppiah P, Thomson WM, Milne T, Yeoh N, Nolan A, et al. The oral microbiome of patients with axial spondyloarthritis compared to healthy individuals. PeerJ. 2016;4:e2095.

(60) Han YW, Ikegami A, Bissada NF, Herbst M, Redline RW, Ashmead GG. Transmission of an uncultivated *bergeyella* strain from the oral cavity to amniotic fluid in a case of preterm birth. J Clin Microbiol. 2006;44:1475-83.

(61) McBain AJ, Bartolo RG, Catrenich CE, Charbonneau D, Ledder RG, Gilbert P. Growth and molecular characterization of dental plaque microcosms. J Appl Microbiol. 2003;94:655-64.

(62) Hooper SJ, Crean SJ, Fardy MJ, Lewis MAO, Spratt DA, Wade WG, et al. A molecular analysis of the bacteria present within oral squamous cell carcinoma. J Med Microbiol. 2007;56:1651-9.

(63) Tran-Hung L, Tran-Thi N, Aboudharam G, Raoult D, Drancourt M. A new method to extract dental pulp DNA: application to universal detection of bacteria. PLoS One. 2007;2:e1062.

(64) Elliott DRF, Walker AW, O'Donovan M, Parkhill J, Fitzgerald RC. A non-endoscopic device to sample the oesophageal microbiota: a case-control study. Lancet Gastroenterol Hepatol. 2017;2:32-42.

(65) Kim KS, Rowlinson MC, Bennion R, Liu C, Talan D, Summanen P, et al. Characterization of *slackia exigua* isolated from human wound infections, including abscesses of intestinal origin. J Clin Microbiol. 2010;48:1070-5.

(66) Kanasi E, Dewhirst FE, Chalmers NI, Kent R,Jr, Moore A, Hughes CV, et al. Clonal analysis of the microbiota of severe early childhood caries. Caries Res. 2010;44:485-97.

(67) Hurley E, Barrett MPJ, Kinirons M, Whelton H, Ryan CA, Stanton C, et al. Comparison of the salivary and dentinal microbiome of children with severe-early childhood caries to the salivary microbiome of caries-free children. BMC Oral Health. 2019;19:13.

(68) Hou J, Zheng H, Li P, Liu H, Zhou H, Yang X. Distinct shifts in the oral microbiota are associated with the progression and aggravation of mucositis during radiotherapy. Radiother Oncol. 2018;129:44-51.

(69) Frandsen EV, Poulsen K, Könönen E, Kilian M. Diversity of *capnocytophaga* species in children and description of *capnocytophaga leadbetteri* sp. nov. and *capnocytophaga* genospecies AHN8471. Int J Syst Evol Microbiol. 2008;58:324-36.

(70) Mussano F, Ferrocino I, Gavrilova N, Genova T, Dell'Acqua A, Cocolin L, et al. Apical periodontitis: preliminary assessment of microbiota by 16S rRNA high throughput amplicon target sequencing. BMC Oral Health. 2018;18:55.

(71) Rusthen S, Kristoffersen AK, Young A, Galtung HK, Petrovski BÉ, Palm Ø, et al. Dysbiotic salivary microbiota in dry mouth and primary Sjögren's syndrome patients. PLoS One. 2019;14:e0218319.

(72) Luo T, Srinivasan U, Ramadugu K, Shedden KA, Neiswanger K, Trumble E, et al. Effects of specimen collection methodologies and storage conditions on the short-term stability of oral microbiome taxonomy. Appl Environ Microbiol. 2016;82:5519-29.

(73) Henne K, Li J, Stoneking M, Kessler O, Schilling H, Sonanini A, et al. Global analysis of saliva as a source of bacterial genes for insights into human population structure and migration studies. BMC Evol Biol. 2014;14:190.

(74) Anesti V, McDonald IR, Ramaswamy M, Wade WG, Kelly DP, Wood AP. Isolation and molecular detection of methylotrophic bacteria occurring in the human mouth. Environ Microbiol. 2005;7:1227-38.

(75) Ikeda E, Shiba T, Ikeda Y, Suda W, Nakasato A, Takeuchi Y, et al. Japanese subgingival microbiota in health *vs* disease and their roles in predicted functions associated with periodontitis. Odontology. 2020;108:280-91.

(76) Valenza G, Veihelmann S, Peplies J, Tichy D, Roldan-Pareja Mdel C, Schlagenhauf U, et al. Microbial changes in periodontitis successfully treated by mechanical plaque removal and systemic amoxicillin and metronidazole. Int J Med Microbiol. 2009;299:427-38.

(77) Han XY, Hong T, Falsen E. *Neisseria bacilliformis* sp. nov. isolated from human infections. J Clin Microbiol. 2006;44:474-9.

(78) Rôças IN, Siqueira JF,Jr. Occurrence of two newly named oral treponemes - *treponema parvum* and *treponema putidum* - in primary endodontic infections. Oral Microbiol Immunol. 2005;20:372-5.

(79) Loy A, Lehner A, Lee N, Adamczyk J, Meier H, Ernst J, et al. Oligonucleotide microarray for 16S rRNA gene-based detection of all recognized lineages of sulfate-reducing prokaryotes in the environment. Appl Environ Microbiol. 2002;68:5064-81.

(80) Ozga AT, Sankaranarayanan K, Tito RY, Obregon-Tito AJ, Foster MW, Tallbull G, et al. Oral microbiome diversity among Cheyenne and Arapaho individuals from Oklahoma. Am J Phys Anthropol. 2016;161:321-7.

(81) Filkins LM, Hampton TH, Gifford AH, Gross MJ, Hogan DA, Sogin ML, et al. Prevalence of streptococci and increased polymicrobial diversity associated with cystic fibrosis patient stability. J Bacteriol. 2012;194:4709-17.

(82) Iwauchi M, Horigome A, Ishikawa K, Mikuni A, Nakano M, Xiao JZ, et al. Relationship between oral and gut microbiota in elderly people. Immun Inflamm Dis. 2019;7:229-36.

(83) Renson A, Jones HE, Beghini F, Segata N, Zolnik CP, Usyk M, et al. Sociodemographic variation in the oral microbiome. Ann Epidemiol. 2019;35:73-80.e2.

(84) Lim Y, Totsika M, Morrison M, Punyadeera C. The saliva microbiome profiles are minimally affected by collection method or DNA extraction protocols. Sci Rep. 2017;7:8523.

(85) Siqueira JF,Jr, Rôças IN. Uncultivated phylotypes and newly named species associated with primary and persistent endodontic infections. J Clin Microbiol. 2005;43:3314-9.

(86) Radhakrishnan P, Anbalagan R, Barani R, Mani M, Seshadri KG, Srikanth P. Sequencing of *porphyromonas gingivalis* from saliva in patients with periodontitis and type 2 diabetes mellitus. Indian J Med Microbiol. 2019;37:54-9.

(87) Dewhirst FE, Paster BJ, Tzellas N, Coleman B, Downes J, Spratt DA, et al. Characterization of novel human oral isolates and cloned 16S rDNA sequences that fall in the family coriobacteriaceae: description of *olsenella* gen. nov., reclassification of *lactobacillus uli* as *olsenella uli* comb. nov. and description of *olsenella profusa* sp. nov. Int J Syst Evol Microbiol. 2001;51:1797-804.

(88) Brands B, Vianna ME, Seyfarth I, Conrads G, Horz HP. Complementary retrieval of 16S rRNA gene sequences using broad-range primers with inosine at the 3'-terminus: implications for the study of microbial diversity. FEMS Microbiol Ecol. 2010;71:157-67.

(89) Vandecandelaere I, Matthijs N, Van Nieuwerburgh F, Deforce D, Vosters P, De Bus L, et al. Assessment of microbial diversity in biofilms recovered from endotracheal tubes using culture dependent and independent approaches. PLoS One. 2012;7:e38401.

(90) Bolivar I, Whiteson K, Stadelmann B, Baratti-Mayer D, Gizard Y, Mombelli A, et al. Bacterial diversity in oral samples of children in niger with acute noma, acute necrotizing gingivitis, and healthy controls. PLoS Negl Trop Dis. 2012;6:e1556.

(91) Vengerfeldt V, Špilka K, Saag M, Preem JK, Oopkaup K, Truu J, et al. Highly diverse microbiota in dental root canals in cases of apical periodontitis (data of illumina sequencing). J Endod. 2014;40:1778-83.

(92) Zhang Y, Liu Y, Ma Q, Song Y, Zhang Q, Wang X, et al. Identification of *lactobacillus* from the saliva of adult patients with caries using matrix-assisted laser desorption/ionization time-of-flight mass spectrometry. PLoS One. 2014;9:e106185.

(93) Niazi SA, Al Kharusi HS, Patel S, Bruce K, Beighton D, Foschi F, et al. Isolation of *propionibacterium acnes* among the microbiota of primary endodontic infections with and without intraoral communication. Clin Oral Investig. 2016;20:2149-60.

(94) Xu J, Xiang C, Zhang C, Xu B, Wu J, Wang R, et al. Microbial biomarkers of common tongue coatings in patients with gastric cancer. Microb Pathog. 2019;127:97-105.

(95) Fujita Y, Iikura M, Horio Y, Ohkusu K, Kobayashi N. Pulmonary *actinomyces graevenitzii* infection presenting as organizing pneumonia diagnosed by PCR analysis. J Med Microbiol. 2012;61:1156-8.

(96) Downes J, Vartoukian SR, Dewhirst FE, Izard J, Chen T, Yu WH, et al. *Pyramidobacter piscolens* gen. nov., sp. nov., a member of the phylum 'synergistetes' isolated from the human oral cavity. Int J Syst Evol Microbiol. 2009;59:972-80.

(97) Leake SL, Pagni M, Falquet L, Taroni F, Greub G. The salivary microbiome for differentiating individuals: proof of principle. Microbes Infect. 2016;18:399-405.

(98) Weyrich LS, Duchene S, Soubrier J, Arriola L, Llamas B, Breen J, et al. Neanderthal behaviour, diet, and disease inferred from ancient DNA in dental calculus. Nature. 2017;544:357-61.

(99) Moon JH, Lee JH, Lee JY. Subgingival microbiome in smokers and non-smokers in Korean chronic periodontitis patients. Mol Oral Microbiol. 2015;30:227-41.

(100) Abusleme L, Dupuy AK, Dutzan N, Silva N, Burleson JA, Strausbaugh LD, et al. The subgingival microbiome in health and periodontitis and its relationship with community biomass and inflammation. ISME J. 2013;7:1016-25.

(101) Shi M, Wei Y, Hu W, Nie Y, Wu X, Lu R. The subgingival microbiome of periodontal pockets with different probing depths in chronic and aggressive periodontitis: a pilot study. Front Cell Infect Microbiol. 2018;8:124.

(102) Zhou M, Rong R, Munro D, Zhu C, Gao X, Zhang Q, et al. Investigation of the effect of type 2 diabetes mellitus on subgingival plaque microbiota by high-throughput 16S rDNA pyrosequencing. PLoS One. 2013;8:e61516.

(103) Heuer W, Kettenring A, Stumpp SN, Eberhard J, Gellermann E, Winkel A, et al. Metagenomic analysis of the peri-implant and periodontal microflora in patients with clinical signs of gingivitis or mucositis. Clin Oral Investig. 2012;16:843-50.

(104) Acharya A, Chen T, Chan Y, Watt RM, Jin L, Mattheos N. Species-level salivary microbial indicators of well-resolved periodontitis: a preliminary investigation. Front Cell Infect Microbiol. 2019;9:347.

(105) Chen H, Liu Y, Zhang M, Wang G, Qi Z, Bridgewater L, et al. A *filifactor alocis*-centered co-occurrence group associates with periodontitis across different oral habitats. Sci Rep. 2015;5:9053.

(106) Xu X, He J, Xue J, Wang Y, Li K, Zhang K, et al. Oral cavity contains distinct niches with dynamic microbial communities. Environ Microbiol. 2015;17:699-710.

(107) Tao D, Li F, Feng X, Wong MCM, Lu H. Plaque biofilm microbial diversity in infants aged 12 months and their mothers with or without dental caries: a pilot study. BMC Oral Health. 2018;18:228.

(108) Li Y, Zou CG, Fu Y, Li Y, Zhou Q, Liu B, et al. Oral microbial community typing of caries and pigment in primary dentition. BMC Genomics. 2016;17:558.

(109) Eren AM, Borisy GG, Huse SM, Mark Welch JL. Oligotyping analysis of the human oral microbiome. Proc Natl Acad Sci U S A. 2014;111:2875.

(110) Gruffaz M, Zhang T, Marshall V, Gonçalves P, Ramaswami R, Labo N, et al. Signatures of oral microbiome in HIV-infected individuals with oral Kaposi's sarcoma and cell-associated KSHV DNA. PLoS Pathog. 2020;16:e1008114.

(111) Kurabayashi H, Kaneko A, Sekiya R, Karakida K, Sasaki M, Nakatogawa N, et al. Identification of oral bacteria by 16S rRNA gene analysis in elderly persons requiring nursing care. J Infect Chemother. 2011;17:40-4.

(112) Sano N, Yamashita Y, Fukuda K, Taniguchi H, Goto M, Miyamoto H. Comprehensive analysis of bacterial flora in postoperative maxillary cyst fluid by 16S rRNA gene and culture methods. ISRN Dent. 2012;2012:840483.

(113) Zhang C, Hou BX, Zhao HY, Sun Z. Microbial diversity in failed endodontic root-filled teeth. Chin Med J (Engl). 2012;125:1163-8.

(114) Süzük Yıldız S, Kaşkatepe B, Altınok S, Çetin M, Karagöz A, Savaş S. Comparison of MALDI-TOF and 16S rRNA methods in identification of viridans group streptococci. Mikrobiyol Bul. 2017;51:1-9.

(115) Lazarevic V, Gaïa N, Girard M, Schrenzel J. Decontamination of 16S rRNA gene amplicon sequence datasets based on bacterial load assessment by qPCR. BMC Microbiol. 2016;16:73.

(116) de Lillo A, Ashley FP, Palmer RM, Munson MA, Kyriacou L, Weightman AJ, et al. Novel subgingival bacterial phylotypes detected using multiple universal polymerase chain reaction primer sets. Oral Microbiol Immunol. 2006;21:61-8.

(117) van der Reijden, W. A., Brunner J, Bosch-Tijhof CJ, van Trappen S, Rijnsburger MC, de Graaff MP, et al. Phylogenetic variation of *aggregatibacter actinomycetemcomitans* serotype e reveals an aberrant distinct evolutionary stable lineage. Infect Genet Evol. 2010;10:1124-31.

(118) Dyrhovden R, Nygaard RM, Patel R, Ulvestad E, Kommedal Ø. The bacterial aetiology of pleural empyema. A descriptive and comparative metagenomic study. Clin Microbiol Infect. 2019;25:981-6.

(119) Segal LN, Alekseyenko AV, Clemente JC, Kulkarni R, Wu B, Gao Z, et al. Enrichment of lung microbiome with supraglottic taxa is associated with increased pulmonary inflammation. Microbiome. 2013;1:19.

(120) Pushalkar S, Mane SP, Ji X, Li Y, Evans C, Crasta OR, et al. Microbial diversity in saliva of oral squamous cell carcinoma. FEMS Immunol Med Microbiol. 2011;61:269-77.

(121) Jiang WX, Hu YJ, Gao L, He ZY, Zhu CL, Ma R, et al. The impact of various time intervals on the supragingival plaque dynamic core microbiome. PLoS One. 2015;10:e0124631.

(122) El-Latif Hesham A, Alrumman SA. Antibacterial activity of Miswak Salvadora persica extracts against isolated and genetically identified oral cavity pathogens. Technol Health Care. 2016;24 Suppl 2:S841-8.

(123) Arul AS, Palanivelu P. Biofilm forming ability of a new bacterial isolate from dental caries: an atomic force microscopic study. J Nat Sci Biol Med. 2014;5:278-83.

(124) Yang SF, Huang HD, Fan WL, Jong YJ, Chen MK, Huang CN, et al. Compositional and functional variations of oral microbiota associated with the mutational changes in oral cancer. Oral Oncol. 2018;77:1-8.

(125) Saleem HG, Seers CA, Sabri AN, Reynolds EC. Dental plaque bacteria with reduced susceptibility to chlorhexidine are multidrug resistant. BMC Microbiol. 2016;16:214.

(126) Perkins SD, Woeltje KF, Angenent LT. Endotracheal tube biofilm inoculation of oral flora and subsequent colonization of opportunistic pathogens. Int J Med Microbiol. 2010;300:503-11.

(127) Costello EK, Carlisle EM, Bik EM, Morowitz MJ, Relman DA. Microbiome assembly across multiple body sites in low-birthweight infants. mBio. 2013;4:782.

(128) Kim YJ, Choi YS, Baek KJ, Yoon SH, Park HK, Choi Y. Mucosal and salivary microbiota associated with recurrent aphthous stomatitis. BMC Microbiol. 2016;16 Suppl 1:57.

(129) Pereira PAB, Aho VTE, Paulin L, Pekkonen E, Auvinen P, Scheperjans F. Oral and nasal microbiota in Parkinson's disease. Parkinsonism Relat Disord. 2017;38:61-7.

(130) Lopes Dos Santos Santiago, G., Brusselle G, Dauwe K, Deschaght P, Verhofstede C, Vaneechoutte D, et al. Influence of chronic azithromycin treatment on the composition of the oropharyngeal microbial community in patients with severe asthma. BMC Microbiol. 2017;17:109.

(131) Nakano Y, Takeshita T, Kamio N, Shiota S, Shibata Y, Suzuki N, et al. Supervised machine learning-based classification of oral malodor based on the microbiota in saliva samples. Artif Intell Med. 2014;60:97-101.

(132) Yeoh YK, Chan MH, Chen Z, Lam EWH, Wong PY, Ngai CM, et al. The human oral cavity microbiota composition during acute tonsillitis: a cross-sectional survey. BMC Oral Health. 2019;19:275.

(133) Wyss C, Choi BK, Schüpbach P, Guggenheim B, Göbel UB. *Treponema maltophilum* sp. nov., a small oral spirochete isolated from human periodontal lesions. Int J Syst Bacteriol. 1996;46:745-52.

(134) Wyss C, Dewhirst FE, Gmür R, Thurnheer T, Xue Y, Schüpbach P, et al. *Treponema parvum* sp. nov., a small, glucoronic or galacturonic acid-dependent oral spirochaete from lesions of human periodontitis and acute necrotizing ulcerative gingivitis. Int J Syst Evol Microbiol. 2001;51:955-62.

(135) Salman HA, Senthilkumar R, Mahmood BS, Imran K. Detection and characterization of *streptococcus downei*, a rare bacterial species of mutans streptococci from caries-active patients. Indian J Dent Res. 2019;30:579-82.

(136) Dewhirst FE, Chen T, Izard J, Paster BJ, Tanner ACR, Yu W, et al. The human oral microbiome. J Bacteriol. 2010;192:5002.

(137) Vartoukian SR, Palmer RM, Wade WG. Diversity and morphology of members of the phylum "synergistetes" in periodontal health and disease. Appl Environ Microbiol. 2009;75:3777-86.

(138) Vickerman MM, Brossard KA, Funk DB, Jesionowski AM, Gill SR. Phylogenetic analysis of bacterial and archaeal species in symptomatic and asymptomatic endodontic infections. J Med Microbiol. 2007;56:110-8.

(139) Hirasawa M, Kurita-Ochia T. Probiotic potential of lactobacilli isolated from saliva of periodontally healthy individuals. Oral Health Prev Dent. 2020;18:563-70.

(140) Silva DG, Tinoco EM, Rocha GA, Rocha AM, Guerra JB, Saraiva IE, et al. *Helicobacter pylori* transiently in the mouth may participate in the transmission of infection. Mem Inst Oswaldo Cruz. 2010;105:657-60.

(141) Collins MD, Love DN, Karjalainen J, Kanervo A, Forsblom B, Willems A, et al. Phylogenetic analysis of members of the genus *porphyromonas* and description of *porphyromonas cangingivalis* sp. nov. and *porphyromonas cansulci* sp. nov. Int J Syst Bacteriol. 1994;44:674-9.

(142) Anderson AC, Sanunu M, Schneider C, Clad A, Karygianni L, Hellwig E, et al. Rapid species-level identification of vaginal and oral lactobacilli using MALDI-TOF MS analysis and 16S rDNA sequencing. BMC Microbiol. 2014;14:312.

(143) Dix K, Watanabe SM, McArdle S, Lee DI, Randolph C, Moncla B, et al. Species-specific oligodeoxynucleotide probes for the identification of periodontal bacteria. J Clin Microbiol. 1990;28:319-23.

(144) Willcox MD, Zhu H, Knox KW. *Streptococcus australis* sp. nov., a novel oral streptococcus. Int J Syst Evol Microbiol. 2001;51:1277-81.

(145) Ciantar M, Newman HN, Wilson M, Spratt DA. Molecular identification of *capnocytophaga* spp. via 16S rRNA PCR-restriction fragment length polymorphism analysis. J Clin Microbiol. 2005;43:1894-1901.
