# Supplementary material for: In silico evaluation and selection of the best 16S rRNA gene primers for use in next-generation sequencing to detect oral bacteria and archaea
Source: Microbiome. 2023 Mar 23;11:58. doi: 10.1186/s40168-023-01481-6 (PMC10035280; doi:10.1186/s40168-023-01481-6)
Supplement: Supplementary file 4 — Additional file 3: List of references from which the archaeal species inhabiting different human-mouth niches were obtained. [file 40168_2023_1481_MOESM3_ESM.docx]

Additional table 4. References from which we obtained the archaeal species inhabiting different human mouth niches.

| **PMID** | **Reference** |
| --- | --- |
| 29138298 | (1) |
| 25950865 | (2) |
| 15067114 | (3) |
| 16597851 | (4) |
| 24859768 | (5) |
| 32167855 | (6) |
| 26557034 | (7) |
| 29401401 | (8) |
| 18707623 | (9) |
| 17172525 | (10) |
| 27166431 | (11) |
| 11267768 | (12) |
| 33024215 | (13) |
| 30796545 | (14) |
| 28273061 | (15) |
| 28648489 | (16) |
| 21539593 | (17) |
| 32690877 | (18) |
| 22561061 | (19) |
| 22326171 | (20) |
| 23078250 | (21) |
| 21338359 | (22) |
| 24025872 | (23) |
| 32925997 | (24) |
| 24320900 | (25) |
| 19572899 | (26) |
| 21420503 | (27) |
| 29797436 | (28) |
| 28826642 | (29) |
| 31666044 | (30) |
| 21253553 | (31) |
| 26194817 | (32) |
| 22897827 | (33) |
| 16186749 | (34) |
| 22827611 | (35) |
| 23201354 | (36) |
| 25633825 | (37) |
| 21541092 | (38) |
| 29907776 | (39) |
| 25797107 | (40) |
| 31372904 | (41) |
| 27926431 | (42) |
| 33016381 | (43) |
| 25074492 | (44) |
| 25830311 | (45) |
| 28623321 | (46) |
| 19702957 | (47) |
| 25708582 | (48) |
| 18326571 | (49) |
| 18757236 | (50) |
| 27010812 | (51) |
| 28609785 | (52) |
| 29497795 | (53) |

**REFERENCES**

(1) Koskinen K, Pausan MR, Perras AK, Beck M, Bang C, Mora M, et al. First insights into the diverse human archaeome: specific detection of archaea in the gastrointestinal tract, lung, and nose and on skin. mBio. 2017;8:e00824-17.

(2) Horz HP. Archaeal lineages within the human microbiome: absent, rare or elusive? Life (Basel). 2015;5:1333-45.

(3) Lepp PW, Brinig MM, Ouverney CC, Palm K, Armitage GC, Relman DA. Methanogenic archaea and human periodontal disease. Proc Natl Acad Sci U S A. 2004;101:6176-81.

(4) Vianna ME, Conrads G, Gomes BP, Horz HP. Identification and quantification of archaea involved in primary endodontic infections. J Clin Microbiol. 2006;44:1274-82.

(5) Li CL, Jiang YT, Liu DL, Qian J, Liang JP, Shu R. Prevalence and quantification of the uncommon archaea phylotype *Thermoplasmata* in chronic periodontitis. Arch Oral Biol. 2014;59:822-8.

(6) Belmok A, de Cena JA, Kyaw CM, Damé-Teixeira N. The oral archaeome: a scoping review. J Dent Res. 2020;99:630-43.

(7) Efenberger M, Agier J, Pawłowska E, Brzezińska-Błaszczyk E. Archaea prevalence in inflamed pulp tissues. Cent Eur J Immunol. 2015;40:194-200.

(8) Brzezińska-Błaszczyk E, Pawłowska E, Płoszaj T, Witas H, Godzik U, Agier J. Presence of archaea and selected bacteria in infected root canal systems. Can J Microbiol. 2018;64:317-26.

(9) Yamabe K, Maeda H, Kokeguchi S, Tanimoto I, Sonoi N, Asakawa S, et al. Distribution of archaea in Japanese patients with periodontitis and humoral immune response to the components. FEMS Microbiol Lett. 2008;287:69-75.

(10) Vickerman MM, Brossard KA, Funk DB, Jesionowski AM, Gill SR. Phylogenetic analysis of bacterial and archaeal species in symptomatic and asymptomatic endodontic infections. J Med Microbiol. 2007;56:110-8.

(11) Huynh HT, Nkamga VD, Signoli M, Tzortzis S, Pinguet R, Audoly G, et al. Restricted diversity of dental calculus methanogens over five centuries, France. Sci Rep. 2016;6:25775.

(12) Kulik EM, Sandmeier H, Hinni K, Meyer J. Identification of archaeal rDNA from subgingival dental plaque by PCR amplification and sequence analysis. FEMS Microbiol Lett. 2001;196:129-33.

(13) Kumpitsch C, Moissl-Eichinger C, Pock J, Thurnher D, Wolf A. Preliminary insights into the impact of primary radiochemotherapy on the salivary microbiome in head and neck squamous cell carcinoma. Sci Rep. 2020;10:16582.

(14) Sogodogo E, Drancourt M, Grine G. Methanogens as emerging pathogens in anaerobic abscesses. Eur J Clin Microbiol Infect Dis. 2019;38:811-8.

(15) Weyrich LS, Duchene S, Soubrier J, Arriola L, Llamas B, Breen J, et al. Neanderthal behaviour, diet, and disease inferred from ancient DNA in dental calculus. Nature. 2017;544:357-61.

(16) Keskin C, Demiryürek EÖ, Onuk EE. Pyrosequencing analysis of cryogenically ground samples from primary and secondary/persistent endodontic infections. J Endod. 2017;43:1309-16.

(17) Matarazzo F, Ribeiro AC, Feres M, Faveri M, Mayer MP. Diversity and quantitative analysis of archaea in aggressive periodontitis and periodontally healthy subjects. J Clin Periodontol. 2011;38:621-7.

(18) Borrel G, Brugère JF, Gribaldo S, Schmitz RA, Moissl-Eichinger C. The host-associated archaeome. Nat Rev Microbiol. 2020;18:622-36.

(19) Horz HP, Seyfarth I, Conrads G. McrA and 16S rRNA gene analysis suggests a novel lineage of archaea phylogenetically affiliated with thermoplasmatales in human subgingival plaque. Anaerobe. 2012;18:373-7.

(20) Mansfield JM, Campbell JH, Bhandari AR, Jesionowski AM, Vickerman MM. Molecular analysis of 16S rRNA genes identifies potentially periodontal pathogenic bacteria and archaea in the plaque of partially erupted third molars. J Oral Maxillofac Surg. 2012;70:1507-14.e1-6.

(21) Nguyen-Hieu T, Khelaifia S, Aboudharam G, Drancourt M. Methanogenic archaea in subgingival sites: a review. APMIS. 2013;121:467-77.

(22) Faveri M, Gonçalves LF, Feres M, Figueiredo LC, Gouveia LA, Shibli JA, et al. Prevalence and microbiological diversity of archaea in peri-implantitis subjects by 16S ribosomal RNA clonal analysis. J Periodontal Res. 2011;46:338-44.

(23) Ashok N, Warad S, Singh VP, Chaudhari H, Narayanan A, Rodrigues J. Prevalence of archaea in chronic periodontitis patients in an Indian population. Indian J Dent Res. 2013;24:289-93.

(24) Aleksandrowicz P, Brzezińska-Błaszczyk E, Dudko A, Agier J. Archaea occurrence in the subgingival biofilm in patients with peri-implantitis and periodontitis. Int J Periodontics Restorative Dent. 2020;40:677-83.

(25) Lira EA, Ramiro FS, Chiarelli FM, Dias RR, Feres M, Figueiredo LC, et al. Reduction in prevalence of archaea after periodontal therapy in subjects with generalized aggressive periodontitis. Aust Dent J. 2013;58:442-7.

(26) Li CL, Liu DL, Jiang YT, Zhou YB, Zhang MZ, Jiang W, et al. Prevalence and molecular diversity of archaea in subgingival pockets of periodontitis patients. Oral Microbiol Immunol. 2009;24:343-6.

(27) Dridi B, Raoult D, Drancourt M. Archaea as emerging organisms in complex human microbiomes. Anaerobe. 2011;17:56-63.

(28) Ramiro FS, de Lira E, Soares G, Retamal-Valdes B, Feres M, Figueiredo LC, et al. Effects of different periodontal treatments in changing the prevalence and levels of archaea present in the subgingival biofilm of subjects with periodontitis: a secondary analysis from a randomized controlled clinical trial. Int J Dent Hyg. 2018;16:569-75.

(29) Moissl-Eichinger C, Pausan M, Taffner J, Berg G, Bang C, Schmitz RA. Archaea are interactive components of complex microbiomes. Trends Microbiol. 2018;26:70-85.

(30) Sogodogo E, Doumbo O, Aboudharam G, Kouriba B, Diawara O, Koita H, et al. First characterization of methanogens in oral cavity in Malian patients with oral cavity pathologies. BMC Oral Health. 2019;19:232.

(31) Horz HP, Conrads G. The discussion goes on: what is the role of euryarchaeota in humans? Archaea. 2010;2010:967271.

(32) Huynh HT, Verneau J, Levasseur A, Drancourt M, Aboudharam G. Bacteria and archaea paleomicrobiology of the dental calculus: a review. Mol Oral Microbiol. 2016;31:234-42.

(33) Dridi B. Laboratory tools for detection of archaea in humans. Clin Microbiol Infect. 2012;18:825-33.

(34) Siqueira JF,Jr, Rôças IN, Baumgartner JC, Xia T. Searching for archaea in infections of endodontic origin. J Endod. 2005;31:719-22.

(35) Matarazzo F, Ribeiro AC, Faveri M, Taddei C, Martinez MB, Mayer MP. The domain archaea in human mucosal surfaces. Clin Microbiol Infect. 2012;18:834-40.

(36) Wade WG. The oral microbiome in health and disease. Pharmacol Res. 2013;69:137-43.

(37) Huynh HT, Nkamga VD, Drancourt M, Aboudharam G. Genetic variants of dental plaque *Methanobrevibacter oralis*. Eur J Clin Microbiol Infect Dis. 2015;34:1097-101.

(38) Horz HP, Conrads G. Methanogenic archaea and oral infections - ways to unravel the black box. J Oral Microbiol. 2011;3:doi:10.3402/jom.v3i0.5940.

(39) Grine G, Terrer E, Boualam MA, Aboudharam G, Chaudet H, Ruimy R, et al. Tobacco-smoking-related prevalence of methanogens in the oral fluid microbiota. Sci Rep. 2018;8:9197.

(40) Horz HP, Robertz N, Vianna ME, Henne K, Conrads G. Relationship between methanogenic archaea and subgingival microbial complexes in human periodontitis. Anaerobe. 2015;35:10-2.

(41) Mosaddad SA, Tahmasebi E, Yazdanian A, Rezvani MB, Seifalian A, Yazdanian M, et al. Oral microbial biofilms: an update. Eur J Clin Microbiol Infect Dis. 2019;38:2005-19.

(42) Sampaio-Maia B, Caldas IM, Pereira ML, Pérez-Mongiovi D, Araujo R. The oral microbiome in health and its implication in oral and systemic diseases. Adv Appl Microbiol. 2016;97:171-210.

(43) Kensara A, Hefni E, Williams MA, Saito H, Mongodin E, Masri R. Microbiological profile and human immune response associated with peri-implantitis: a systematic review. J Prosthodont. 2020;doi: 10.1111/jopr.13270.

(44) Pérez-Chaparro PJ, Gonçalves C, Figueiredo LC, Faveri M, Lobão E, Tamashiro N, et al. Newly identified pathogens associated with periodontitis: a systematic review. J Dent Res. 2014;93:846-58.

(45) Huynh HT, Pignoly M, Nkamga VD, Drancourt M, Aboudharam G. The repertoire of archaea cultivated from severe periodontitis. PLoS One. 2015;10:e0121565.

(46) Deng ZL, Szafrański SP, Jarek M, Bhuju S, Wagner-Döbler I. Dysbiosis in chronic periodontitis: key microbial players and interactions with the human host. Sci Rep. 2017;7:3703.

(47) Vianna ME, Conrads G, Gomes BP, Horz HP. T-RFLP-based mcrA gene analysis of methanogenic archaea in association with oral infections and evidence of a novel *Methanobrevibacter* phylotype. Oral Microbiol Immunol. 2009;24:417-22.

(48) Nkamga VD, Huynh HT, Aboudharam G, Ruimy R, Drancourt M. Diversity of human-associated *Methanobrevibacter smithii* isolates revealed by multispacer sequence typing. Curr Microbiol. 2015;70:810-5.

(49) Vianna ME, Holtgraewe S, Seyfarth I, Conrads G, Horz HP. Quantitative analysis of three hydrogenotrophic microbial groups, methanogenic archaea, sulfate-reducing bacteria, and acetogenic bacteria, within plaque biofilms associated with human periodontal disease. J Bacteriol. 2008;190:3779-85.

(50) Conway de Macario E, Macario AJ. Methanogenic archaea in health and disease: a novel paradigm of microbial pathogenesis. Int J Med Microbiol. 2009;299:99-108.

(51) Khelaifia S, Lagier JC, Nkamga VD, Guilhot E, Drancourt M, Raoult D. Aerobic culture of methanogenic archaea without an external source of hydrogen. Eur J Clin Microbiol Infect Dis. 2016;35:985-91.

(52) Philips A, Stolarek I, Kuczkowska B, Juras A, Handschuh L, Piontek J, et al. Comprehensive analysis of microorganisms accompanying human archaeological remains. Gigascience. 2017;6:1-13.

(53) Chaudhary PP, Conway PL, Schlundt J. Methanogens in humans: potentially beneficial or harmful for health. Appl Microbiol Biotechnol. 2018;102:3095-104.
