# Supplementary material for: In silico evaluation and selection of the best 16S rRNA gene primers for use in next-generation sequencing to detect oral bacteria and archaea
Source: Microbiome. 2023 Mar 23;11:58. doi: 10.1186/s40168-023-01481-6 (PMC10035280; doi:10.1186/s40168-023-01481-6)
Supplement: Supplementary file 11 — Additional file 10: Information related to the coverage analysis of the 16S rRNA gene individual primers. [file 40168_2023_1481_MOESM10_ESM.docx]

Additional file 10. Information related to the coverage analysis of the 16S rRNA gene individual primers.

**MATERIAL AND METHODS**

***Allocation of individual primers to the corresponding 16S rRNA gene regions***

The location of the first and last nucleotides of each primer within each sequence with a match was calculated and the mode values for these positions were determined. If there was more than one mode for a position, we chose the one closest to the mean position value. As all the sequences in the two databases were aligned with the 16S rRNA *E. coli* gene, the mode values obtained for each primer enabled us to allocate them to one of the gene regions defined for that organism by Baker *et al*. (1). The reference sequence utilised had 1542 bps distributed in 10 conserved (C1-C10) and nine hypervariable regions (V1-V9).

***Analysis of the coverage of individual primers***

The script used for the VC analysis stored one xlsx file for each evaluated primer. In addition, the analysis produced a summary file in the same format as that which synthesised the results obtained for all the primers. Each line contained the information for each primer. The first columns included the primer identifier, direction, sequence, and length. These were followed by the number of allowed mismatches, the VC (%), the number of analysed sequences, and the number of sequences with and without matches. The file also included the mean and the mode positions of the first and last primer nucleotides in all the corresponding sequences, as well as the 16S rRNA gene region assigned to the initial and the end mode positions. Although we did not include any mismatches, the developed script allowed us to indicate the maximum number permitted.

The SC analysis used two Excel files for each evaluated primer: one with all the species for which at least one genomic variant matched the primer, and another with the non-matched or non-covered species. Again, a file in Excel format summarised the results obtained for all the primers assessed. The first five lines of the file were the same as those obtained from the VC analysis but only included: the SC (%), the number of analysed species, and the number of species with and without matches.

**RESULTS**

**Evaluation of 16S rRNA gene individual primers for the detection of oral bacteria, archaea and both domains**

*Bacterial-specific individual primers*

A total of 302 different individual primers (133 F, 169 R) had some coverage value for detecting oral bacteria (Additional table 9), while 67 (42 F and 25 R) provided no VC or SC. Fifty-nine primers (27 F, 32 R) localised in the gene regions 3, 4, 5, 6, 7, and 9 had bacterial SC values ≥95.00%. Thirteen of them (9 F, 4 R) had archaeal SC values of 0.00%. These bacteria-specific primers belonged to regions 3 and 7 and corresponded to: KP_F044, 046, 047, 048; OP_F048, 050, 096, 108, 116; KP_R018, 020; and OP_R054 and 116.

Additional table 9. Individual primers within a particular bacterial coverage range in each 16S rRNA gene region.

|  | 16S rRNA gene region | | | | | | | | | | | | | | | | | | | |
| --- | --- | --- | --- | --- | --- | --- | --- | --- | --- | --- | --- | --- | --- | --- | --- | --- | --- | --- | --- | --- |
| Coverage (%) | 1 | | 2 | | 3 | | 4 | | 5 | | 6 | | 7 | | 8 | | 9 | | 10 | |
|  | V | S | V | S | V | S | V | S | V | S | V | S | V | S | V | S | V | S | V | S |
| ≥95 | 0 | 0 | 0 | 0 | 20 | 15 | 11 | 19 | 8 | 15 | 1 | 1 | 5 | 6 | 1 | 0 | 0 | 3 | 0 | 0 |
| ≥90 - <95 | 0 | 0 | 0 | 0 | 1 | 4 | 20 | 13 | 23 | 1 | 10 | 10 | 6 | 3 | 0 | 0 | 0 | 5 | 0 | 0 |
| ≥85 - <90 | 0 | 0 | 0 | 0 | 0 | 2 | 4 | 1 | 1 | 15 | 1 | 3 | 4 | 1 | 0 | 1 | 2 | 0 | 0 | 0 |
| ≥80 - <85 | 0 | 6 | 0 | 0 | 1 | 0 | 0 | 1 | 0 | 0 | 3 | 3 | 1 | 3 | 1 | 0 | 0 | 0 | 0 | 0 |
| ≥75 - <80 | 0 | 11 | 0 | 0 | 0 | 0 | 0 | 0 | 0 | 0 | 12 | 0 | 0 | 2 | 0 | 0 | 0 | 2 | 0 | 2 |
| <75% | 44 | 27 | 8 | 8 | 8 | 9 | 9 | 10 | 17 | 18 | 19 | 29 | 13 | 14 | 2 | 3 | 13 | 5 | 33 | 31 |
| Total | 44 | 44 | 8 | 8 | 30 | 30 | 44 | 44 | 49 | 49 | 46 | 46 | 29 | 29 | 4 | 4 | 15 | 15 | 33 | 33 |

V= variant; S= species.

*Archaeal-specific individual primers*

One hundred and seventy-four individual primers (63 F, 111 R) had some coverage value for detecting oral archaea (Additional table 10). Conversely, 195 (112 F, 83 R) had an archaeal VC and SC of 0.00%. Thirty-three primers (7 F, 26 R) covered at least 95.00% of the oral-associated archaeal species in our database and were localised in gene regions 3, 5, 6 and 9 (Additional table 10). Of these, only KP_F016, KP_F018, KP_R006, and KP_R013 were specific to the archaea domain, with bacterial SC values of 0.00%. The two F primers belonged to gene region 3 and the two R to region 6.

Additional table 10. Individual primers within a particular archaeal coverage range in each 16S rRNA gene region.

|  | 16S rRNA gene region | | | | | | | | | | | | | | | | | | | |
| --- | --- | --- | --- | --- | --- | --- | --- | --- | --- | --- | --- | --- | --- | --- | --- | --- | --- | --- | --- | --- |
| Coverage (%) | 1 | | 2 | | 3 | | 4 | | 5 | | 6 | | 7 | | 8 | | 9 | | 10 | |
|  | V | S | V | S | V | S | V | S | V | S | V | S | V | S | V | S | V | S | V | S |
| ≥95 | 0 | 0 | 0 | 0 | 0 | 7 | 0 | 0 | 0 | 14 | 0 | 3 | 0 | 0 | 0 | 0 | 0 | 9 | 0 | 0 |
| ≥90 - <95 | 0 | 0 | 0 | 0 | 0 | 0 | 0 | 0 | 0 | 3 | 0 | 4 | 0 | 0 | 0 | 0 | 0 | 9 | 0 | 0 |
| ≥85 - <90 | 0 | 0 | 0 | 0 | 6 | 2 | 0 | 0 | 1 | 3 | 0 | 1 | 0 | 2 | 0 | 0 | 0 | 1 | 0 | 0 |
| ≥80 - <85 | 0 | 0 | 0 | 0 | 0 | 1 | 0 | 0 | 19 | 0 | 7 | 3 | 0 | 0 | 0 | 0 | 9 | 0 | 0 | 0 |
| ≥75 - <80 | 0 | 0 | 0 | 0 | 3 | 2 | 0 | 0 | 0 | 0 | 0 | 0 | 0 | 0 | 0 | 0 | 0 | 0 | 0 | 0 |
| <75% | 14 | 14 | 1 | 1 | 39 | 36 | 1 | 1 | 20 | 20 | 25 | 21 | 2 | 0 | 0 | 0 | 20 | 10 | 7 | 7 |
| Total | 14 | 14 | 1 | 1 | 48 | 48 | 1 | 1 | 40 | 40 | 32 | 32 | 2 | 2 | 0 | 0 | 29 | 29 | 7 | 7 |

V= variant; S= species.

*Bacterial and archaeal individual primers*

Nineteen different primers (4 F, 15 R), from gene regions 3, 5, and 9, had SC values ≥95.00% simultaneously in the two databases. The individual F and R primers with the best values in both domains were OP_F066 (bacterial SC= 99.48%, archaeal SC= 99.48%) and KP_R031 (bacterial SC= 99.35%, archaeal SC= 98.97%).

**DISCUSSION**

**Comparative analysis of our coverage results of 16S rRNA gene primers with the literature**

*Bacteria-specific individual primers*

Additional table 11 compares the results on individual primers analysed in both our research and the studies mentioned above. It is clear that our estimates of bacterial SC are similar to those of the other research, with differences no greater than 3.29% for: KP_F031; KP_F047; KP_R034; OP_R054; KP_F056; and KP_R053. The latter two primers achieved the highest overall coverage and specificity for bacteria in the study by Klindworth *et al*. (2); in our research, the best-performing primers for the bacteria domain were OP_F116, KP_F048 or KP_R020 (bacterial SC in order = 98.70%, 98,05%, 98,05%; archaeal SC= 0.00%). Moreover, while KP_R053 was also analysed by Ku *et al*. (3), the coverage values they obtained were poorer than those of both Klindworth and in our study (Additional table 11); on the other hand, our bacterial coverage estimates for KP_F032, KP_F049, KP_R040 and KP_R075 were worse (Additional table 11). Nonetheless, the archaeal coverage of the latter primer in our database suggests it would be a good option for detecting both archaea and bacteria.

*Archaea-specific individual primers*

Concerning the archaeal coverage of individual primers, our coverage values were higher than those in the literature for KP_R003, KP_R005 and KP_F083, and similar for KP_F017 and KP_F082 (Additional table 11). Klindworth *et al*. (2) found that KP_F082 and KP_F083, which were previously regarded as targeting both bacteria and archaea, actually only targeted the latter. Although we agree with this about KP_F083, KP_F082 had a bacterial SC herein of 26.40%, which is more than four times higher than the coverage achieved by Klindworth. In any case, because of its poor bacterial coverage, we would not recommend these individual primers as a suitable option for only evaluating oral archaeal species, or for studying the bacterial or archaeal domains together.

Finally, we confirmed the results obtained in other studies for KP_F020, OP_R014 and KP_R035, which are regarded as suitable for detecting both bacteria and archaea (Additional table 11). Although we found that OP_F014 and KP_F078 achieved reasonable archaeal SC, this was lower than the coverage described previously (Additional table 11). Conversely, our bacterial estimates for KP_F078, and the bacterial and archaeal coverage of KP_R038, are better than those reported by Klindworth (2).

Additional table 11. Coverage values obtained from the literature for the individual primers analysed in the present study.

| Present study | Other studies | Results of the present study | | Results of the other studies | | Ref. |
| --- | --- | --- | --- | --- | --- | --- |
| Primer identifier | Primer name | Bacterial SC (%) | Archaeal SC (%) | Bacterial  coverage (%) | Archaeal  coverage (%) |  |
| KP_F031/OP_F023, 024, 034 | 8F | 66.19 | 0.00 | 62.90 | - | (3) |
| KP_F032/OP_F040 | 27F | 75.81 | 0.00 | 12.90 ^e^ | - | (4) |
| KP_F047/OP_F035 | 341F | 96.10 | 0.00 | 98.35 | 84.56 | (5) |
| KP_F049/OP_F038 | 347F | 88.43 | 0.00 | 93.60^a^ | - | (6) |
|  |  |  |  | 91.10^bc^; 90.40^bd^ | - |  |
| KP_F056/OP_F083 | S-D-Bact-0564-a-S-15 | 98.31 | 8.76 | 96.00 | 16.30 | (2) |
| KP_R034/OP_R039 | 803R | 94.80 | 5.67 | 95.40^a^ | - | (6) |
|  |  |  |  | 91.80^bc^;84.90^bd^ | - |  |
| KP_R040 | 907R/926R | 90.9 | 0.00 | ~1.50 ^e^ | - | (4) |
| KP_R053/OP_R062 | 1061R | 96.62 | 3.61 | 75.80 | - | (3) |
|  | S-D-Bact-1061-a-A-17 |  |  | 96.40 | 2.90 | (2) |
| OP_R054 | 338R | 96.10 | 0.00 | ~3.50 ^e^ | - | (4) |
| KP_F017/OP_F001 | 344F | 0.00 | 74.23 | - | 73.20 | (7) |
| KP_F082 | S-*-Univ-0789-a-S-18 | 26.40 | 88.66 | 6.80 | 86.10 | (2) |
| KP_F083 | S-*-Univ-0906-a-S-17 | 3.38 | 96.39 | 0.30 | 83.70 | (2) |
| KP_R003 | S-D-Arch-0519-a-A-19 | 10.27 | 98.97 | 0.10 | 91.30 | (2) |
| KP_R005/OP_R005, 061 | S-D-Arch-0786-a-A-20 | 26.14 | 97.94 | 7.80 | 87.40 | (2) |
| KP_F020/OP_F007 | 519F | 96.49 | 99.48 | 98.00 | 98.20 | (7) |
| KP_F078/OP_F005, 022 | S-*-Univ-0515-a-S-19 | 96.49 | 63.92 | 54.50 | 95.40 | (2) |
| KP_F081 | S-*-Univ-0779-a-S-20 | 0.00 | 0.00 | 0.00 | 0.00 | (2) |
| OP_F014/OP_F047 | 515F | 96.49 | 89.18 | 98.48 | 97.79 | (5) |
| KP_R035/OP_R035 | 805R | 97.79 | 98.97 | 98.17 | 98.40 | (5) |
| KP_R038 | S-D-Bact-0787-b-A-20 | 97.53 | 97.94 | 89.90 | 90.60 | (2) |
| KP_R075 | 1390R/1406R | 94.41 | 96.91 | ~1.00 ^e^ | - | (4) |
| OP_R014/OP_R120 | 806R | 97.53 | 98.45 | 97.50 | 98.43 | (5) |

The coverage values from the other investigations are those obtained when no mismatches were accepted.

a= foregut database; b= Ribosomal Database Project (RDP); c= species coverage; d= sequence coverage; e= non-coverage percentage; F= forward; KP= Klindworth primer; OP= oral primer; R= reverse; Ref.= references; SC= coverage at the species level.

**REFERENCES**

(1) Baker GC, Smith JJ, Cowan DA. Review and re-analysis of domain-specific 16S primers. J Microbiol Methods. 2003;55:541–55.

(2) Klindworth A, Pruesse E, Schweer T, Peplies J, Quast C, Horn M, et al. Evaluation of general 16S ribosomal RNA gene PCR primers for classical and next-generation sequencing-based diversity studies. Nucleic Acids Res. 2013;41:e1.

(3) Ku HJ, Lee JH. Development of a novel long-range 16S rRNA universal primer set for metagenomic analysis of gastrointestinal microbiota in newborn infants. J Microbiol Biotechnol. 2014;24:812–22.

(4) Mao DP, Zhou Q, Chen CY, Quan ZX. Coverage evaluation of universal bacterial primers using the metagenomic datasets. BMC Microbiol. 2012;12:66.

(5) Wasimuddin SK, Ronchi F, Leib SL, Erb M, Ramette A. Evaluation of primer pairs for microbiome profiling from soils to humans within the one health framework. Mol Ecol Resour. 2020;20:1558–71.

(6) Nossa CW, Oberdorf WE, Yang L, Aas JA, Paster BJ, Desantis TZ, et al. Design of 16S rRNA gene primers for 454 pyrosequencing of the human foregut microbiome. World J Gastroenterol. 2010;16:4135–44.

(7) Pausan MR, Csorba C, Singer G, Till H, Schöpf V, Santigli E, et al. Exploring the archaeome: detection of archaeal signatures in the human body. Front Microbiol. 2019;10:2796.
