# Supplementary material for: In silico evaluation and selection of the best 16S rRNA gene primers for use in next-generation sequencing to detect oral bacteria and archaea
Source: Microbiome. 2023 Mar 23;11:58. doi: 10.1186/s40168-023-01481-6 (PMC10035280; doi:10.1186/s40168-023-01481-6)
Supplement: Supplementary file 15 — Additional file 14: Coverage at the variant level of the selected primer pairs for detecting oral bacteria or/and archaea. [file 40168_2023_1481_MOESM14_ESM.docx]

Additional file 14. Coverage at the variant level of the selected primer pairs for detecting oral bacteria or/and archaea.

Additional table 23. Selected primer pairs for detecting oral bacteria in different amplicon-length categories.

|  | | Bacteria | | | | | Archaea | | | | |
| --- | --- | --- | --- | --- | --- | --- | --- | --- | --- | --- | --- |
| LC  (bp) | Primer pair | Gene region | VC (%) | Covered | Not covered | Mean length | Gene region | VC (%) | Covered | Not covered | Mean length |
| 100-300 | KP_F048-OP_R043 | 3-4 | 97.23 | 216964 | 6179 | 183 | - | 0.00 | 0 | 2842 | 0 |
|  | OP_F098-OP_R119 | 4-5 | 93.01 | 207553 | 15590 | 289 | - | 0.00 | 0 | 2842 | 0 |
|  | OP_F066-KP_R040 | 5-6 | 91.69 | 204593 | 18550 | 142 | - | 0.00 | 0 | 2842 | 0 |
|  | OP_F009-OP_R030 | 5-7 | 93.10 | 207745 | 15398 | 297 | - | 0.00 | 0 | 2842 | 0 |
|  | OP_F101-OP_R030 | 6-7 | 90.36 | 201643 | 21500 | 164 | - | 0.00 | 0 | 2842 | 0 |
|  | KP_F061-KP_R074 | 6-7 | 92.27 | 205896 | 17247 | 206 | - | 0.00 | 0 | 2842 | 0 |
| 301-600 | KP_F048-KP_R031 | 3-5 | 96.99 | 216423 | 6720 | 455 | - | 0.00 | 0 | 2842 | 0 |
|  | KP_F048-OP_R073 | 3-6 | 96.33 | 214952 | 8191 | 547 | - | 0.00 | 0 | 2842 | 0 |
|  | KP_F048-OP_R050 | 3-6 | 91.51 | 204199 | 18944 | 579 | - | 0.00 | 0 | 2842 | 0 |
|  | KP_F051-KP_R041 | 4-6 | 91.65 | 204521 | 18622 | 411 | - | 0.00 | 0 | 2842 | 0 |
|  | KP_F051-OP_R030 | 4-7 | 96.68 | 215732 | 7411 | 566 | - | 0.00 | 0 | 2842 | 0 |
|  | OP_F116-KP_R060 | 7-9 | 33.29 | 74277 | 148866 | 308 | - | 0.00 | 0 | 2842 | 0 |
| >600 | KP_F048-OP_R030 | 3-7 | 96.68 | 215742 | 7401 | 733 | - | 0.00 | 0 | 2842 | 0 |
|  | KP_F048-KP_R074 | 3-7 | 97.28 | 217066 | 6077 | 767 | - | 0.00 | 0 | 2842 | 0 |
|  | KP_F048-KP_R060 | 3-9 | 33.21 | 74098 | 149045 | 1061 | - | 0.00 | 0 | 2842 | 0 |
|  | KP_F056-KP_R077 | 4-9 | 28.24 | 63010 | 160133 | 845 | - | 0.00 | 0 | 2842 | 0 |

bp= base pair; LC= length category; VC= coverage at the variant level.

VC was estimated as the number of matches divided by the number of ASVs in the database (223143 in the oral-bacteria and 2842 in the oral-archaea).

Additional table 24. Selected primer pairs for detecting oral archaea in different amplicon-length categories.

|  | | Bacteria | | | | | Archaea | | | | |
| --- | --- | --- | --- | --- | --- | --- | --- | --- | --- | --- | --- |
| LC  (bp) | Primer pair | Gene region | VC (%) | Covered | Not covered | Mean length | Gene region | VC (%) | Covered | Not covered | Mean length |
| 100-300 | KP_F018-KP_R002 | - | 0.00 | 0 | 223143 | 0 | 3 | 82.44 | 2343 | 499 | 144 |
|  | KP_F016-KP_R003 | - | 0.00 | 0 | 223143 | 0 | 3 | 64.39 | 1830 | 1012 | 158 |
|  | OP_F066-KP_R013 | - | 0.00 | 0 | 223143 | 0 | 5-6 | 82.93 | 2357 | 485 | 275 |
| 301-600 | KP_F018-KP_R032 | - | 0.00 | 0 | 223143 | 0 | 3-5 | 81.84 | 2326 | 516 | 414 |
|  | KP_F018-OP_R073 | - | 0.00 | 0 | 223143 | 0 | 3-5 | 81.25 | 2309 | 533 | 510 |
|  | KP_F020-KP_R013 | - | 0.00 | 0 | 223143 | 0 | 3-6 | 83.08 | 2361 | 481 | 542 |
|  | OP_F114-KP_R007 | - | 0.00 | 0 | 223143 | 0 | 3-6 | 81.84 | 2326 | 516 | 557 |
|  | KP_F022-OP_R016 | - | 0.00 | 0 | 223143 | 0 | 5-9 | 80.82 | 2297 | 545 | 490 |
|  | KP_F022-KP_R063 | - | 0.00 | 0 | 223143 | 0 | 5-9 | 71.22 | 2024 | 818 | 585 |
| >600 | OP_F114-KP_R013 | - | 0.00 | 0 | 223143 | 0 | 3-6 | 82.90 | 2356 | 486 | 679 |
|  | KP_F018-KP_R063 | - | 0.00 | 0 | 223143 | 0 | 3-9 | 71.53 | 2033 | 809 | 1118 |
|  | KP_F016-KP_R063 | - | 0.00 | 0 | 223143 | 0 | 3-9 | 56.86 | 1616 | 1226 | 1128 |
|  | OP_F066-OP_R016 | - | 0.00 | 0 | 223143 | 0 | 5-9 | 81.84 | 2326 | 516 | 624 |

bp= base pair; LC= length category; VC= coverage at the variant level.

VC was estimated as the number of matches divided by the number of ASVs in the database (223143 in the oral-bacteria and 2842 in the oral-archaea).

Additional table 25. Selected primer pairs for simultaneously detecting oral bacteria and archaea in different amplicon-length categories.

|  | | Bacteria | | | | | Archaea | | | | |
| --- | --- | --- | --- | --- | --- | --- | --- | --- | --- | --- | --- |
| LC  (bp) | Primer pair | Gene region | VC (%) | Covered | Not covered | Mean length | Gene region | VC (%) | Covered | Not covered | Mean length |
| 100-300 | OP_F114-KP_R002 | 3-4 | 93.70 | 209093 | 14050 | 188 | 3 | 84.27 | 2395 | 447 | 152 |
|  | KP_F020-KP_R032 | 4-5 | 93.53 | 208705 | 14438 | 284 | 3-5 | 83.99 | 2387 | 455 | 285 |
|  | OP_F066-OP_R073 | 5-6 | 96.58 | 215520 | 7623 | 110 | 5 | 82.30 | 2339 | 503 | 114 |
| 301-600 | OP_F114-KP_R031 | 3-5 | 96.94 | 216306 | 6837 | 457 | 3-5 | 83.57 | 2375 | 467 | 422 |
|  | OP_F114-OP_R073 | 3-6 | 96.28 | 214835 | 8308 | 549 | 3-5 | 82.30 | 2339 | 503 | 518 |
|  | KP_F020-OP_R073 | 4-6 | 92.87 | 207226 | 15917 | 376 | 3-5 | 82.44 | 2343 | 499 | 381 |
| >600 | OP_F114-OP_R121 | 3-9 | 33.34 | 74389 | 148754 | 1062 | 3-9 | 82.83 | 2354 | 488 | 1035 |
|  | KP_F020-OP_R121 | 4-9 | 33.02 | 73673 | 149470 | 888 | 3-9 | 82.93 | 2357 | 485 | 897 |
|  | OP_F066-OP_R121 | 5-9 | 33.47 | 74691 | 148452 | 622 | 5-9 | 82.83 | 2354 | 488 | 630 |

bp= base pair; LC= length category; VC= coverage at the variant level.

VC was estimated as the number of matches divided by the number of ASVs in the database (223143 in the oral-bacteria and 2842 in the oral-archaea).
